# Aversive Learning of Colored Lights in Walking Honeybees

**DOI:** 10.3389/fnbeh.2017.00094

**Published:** 2017-05-22

**Authors:** Nicholas H. Kirkerud, Ulrike Schlegel, C. Giovanni Galizia

**Affiliations:** ^1^Neurobiology, University of KonstanzKonstanz, Germany; ^2^International Max-Planck Research School for Organismal Biology, University of KonstanzKonstanz, Germany; ^3^Department of Biosciences, University of OsloOslo, Norway

**Keywords:** visual learning, aversive operant conditioning, relief learning, honeybee, discriminatory fear learning, signaled active avoidance

## Abstract

The honeybee has been established as an important model organism in studies on visual learning. So far the emphasis has been on appetitive conditioning, simulating floral discrimination, and homing behavior, where bees perform exceptionally well in visual discrimination tasks. However, bees in the wild also face dangers, and recent findings suggest that what is learned about visual percepts is highly context dependent. A stimulus that follows an unpleasant period, is associated with the feeling of relief- or safety in humans and animals, thus acquiring a positive meaning. Whether this is also the case in honeybees is still an open question. Here, we conditioned bees aversively in a walking arena where each half was illuminated by light of a specific wavelength and intensity, one of which was combined with electric shocks. In this paradigm, the bees' preferences to the different lights were modified through nine conditioning trials, forming robust escape, and avoidance behaviors. Strikingly, we found that while 465 nm (human blue) and 590 nm (human yellow) lights both could acquire negative valences (inducing avoidance response), 525 nm (human green) light could not. This indicates that green light holds an innate meaning of safety which is difficult to overrule even through intensive aversive conditioning. The bees had slight initial preferences to green over the blue and the yellow lights, which could be compensated by adjusting light intensity. However, this initial bias played a minor role while the chromatic properties were the most salient characteristics of the light stimuli during aversive conditioning. Moreover, bees could learn the light signaling safety, revealing the existence of a relief component in aversive operant conditioning, similar to what has been observed in other animals.

## Introduction

Honeybees have a well-developed visual system which has been subjected to numerous studies since Karl von Frisch demonstrated early in the previous century that they can learn colors (Frisch, 1914). Bees have evolved color vision which share basic principles with primate color vision, but is shifted toward shorter wavelengths (Backhaus, 1992). They depend on color vision to discriminate between flowers, and probably also to detect danger while foraging. Color discrimination learning has been extensively studied in either harnessed or freely flying honeybees. In a harnessed state, both appetitive and aversive classical (pavlovian) conditioning with colors have been successfully demonstrated (Proboscis and Sting Extension Reflex, respectively). In the appetitive paradigm honeybees quickly learn a sucrose reward, but show only weak color discrimination abilities, for which ablation of antennae is necessary (Hori et al., 2006; Niggebrugge et al., 2009). However, intact Africanized honeybees learn to discriminate between blue, green and violet lights in a proboscis extension reflex assay, but fail to learn the violet light when this is rewarded (Jernigan et al., 2014). In an aversive paradigm, harnessed bees can learn to discriminate between visual stimuli based on both chromatic and achromatic (intensity) properties, without the necessity of antennal deprivation (Mota et al., 2011b). Furthermore, colors can set the occasion for odor discrimination by providing context without being directly associated with the reward in PER (Mota et al., 2011a; Plath et al., 2012). Free flying bees show strong discrimination learning to colors in appetitive conditioning (Frisch, 1914; Menzel, 1967; Dyer and Chittka, 2004; Giurfa, 2004). However, the behavior toward the unreinforced color (λ^−^) is not modified, unless this color is sufficiently similar to the reinforced one (λ^+^), in which case generalization occurs. In a study where λ^+^ was awarded with sucrose solution while λ^−^ was punished with quinine solution, bees generalized less for similar wavelengths than when λ^−^ was paired with water, indicating that the addition of an aversive component facilitated color discrimination (Avargues-Weber et al., 2010). Honey bees that were operantly conditioned to discriminate a shock-paired color from a safe color in a walking assay displayed passive avoidance of the shock-paired color, while yoked bees (deprived of control over the shocks) exhibited learned helplessness, a well-studied phenomenon present in a range of other species, including humans (Dinges et al., 2017). However, because aversive operant paradigms using visually discriminative stimuli are uncommon, what the bees learn, and how they learn it in such paradigms, is largely uncharted territory.

Depending on stimulus timing, a single reinforcement type like electric shocks can result in the conditioned stimulus acquiring either a negative or positive valence, which in humans and rodents have been shown to involve both the fear and the reward-pathway of the brain (Seymour et al., 2005; Andreatta et al., 2012; Diegelmann et al., 2013; Gerber et al., 2014). This is thought to be a result of the “pain-relief” experienced after the cessation of an unpleasant stimulus (Konorski, 1948). Alternatively, any stimulus presented in a context where the animal is in imminent danger, which does not overlap or directly predict the punishment, could acquire a positive valence: safety-learning (review by Kong et al., 2014). A relief-component in visual classical conditioning with electric shocks was recently demonstrated in *Drosophila* (Vogt et al., 2015). Whether honeybees also experience relief-learning is still unknown, however, it has been shown with backward appetitive conditioning with odors that the CS (Conditioned Stimulus) can acquire inhibitory properties (Hellstern et al., 1998; Felsenberg et al., 2014). This argues for the possibility that a similar property could be established also for a stimulus signaling safety in aversive differential conditioning. Whether insects are capable of acquiring specific memories to parallel stimuli predicting danger and safety remains to be investigated.

We recently developed an apparatus for automatic conditioning of free walking bees, Automatic Performance Index System (APIS), and established an aversive learning paradigm with olfactory cues (Kirkerud et al., 2013). In the present study, we introduced an aversive operant visual learning paradigm for honeybees in the same apparatus. With this paradigm, we investigated whether bees learn to avoid light stimuli of different colors and intensities that predict danger (electric shocks) and approach lights that predict safety. We found that bees trained in the aversive learning paradigm changed their responses to light stimuli in an adaptive way to avoid electric shocks. The valence of either light stimulus (signaling danger or safety) was subjected to modification, indicating the presence of a relief-component. Furthermore, the learned avoidance was strong enough to overrule natural phototaxis. Our results indicate specific tendencies in learnability toward the different colors. In particular, bees learned to avoid both the blue and yellow (for humans) light when either of these signaled danger, but failed to learn to avoid the green light when this served as λ^+^. Bees showed an initial preference to green (vs. blue and yellow) which could be subdued by adjusting for relative intensity levels. Moreover, our data reveal that relative intensity played a minor role compared to the wavelengths of the lights during aversive learning. Indeed, inability to associate the green light with danger persisted when intensities of the lights were adjusted to levels that produced equal initial preferences. We propose that honeybee foragers regard green light as a safe stimulus due to innate and/or acquired positive valence which is difficult to overrule. When considered in context with findings from previous studies which employed different color learning paradigms, our results are compatible with a view of top-down modulation of color learning where central processes in the brain regulate color perception based on the situation in which the animal finds itself. However, this would need dedicated experiments, including pharmacological manipulations, and a more thorough analysis of the role green light and other colors plays in different aversive and appetitive conditioning paradigms.

## Materials and methods

Experiments I-II were conducted on bees from 4 different queen-right hives being kept outdoor at the roof of the University of Konstanz (Germany; between July and October 2015). For experiments III-IV we used bees from an indoor queen-right hive (about 1 m^3^ meshed wooden cage, 21–25°C, 30–60% humidity, light-darkness-cycles: 14/10 h, including UV light; between November and December 2015). Outbound honeybee foragers (*Apis mellifera*) were individually caught from the hive entrance with customized Falcon® tubes and directly released into the conditioning chamber within 10 min after capture, without feeding them. Bees were given 2 min to habituate in the chamber before conditioning protocols were started. Each bee was subjected to a single conditioning protocol, and sacrificed in 70% ethanol directly after the protocol ended.

### APIS-automatic performance index system

**Figure 1:**
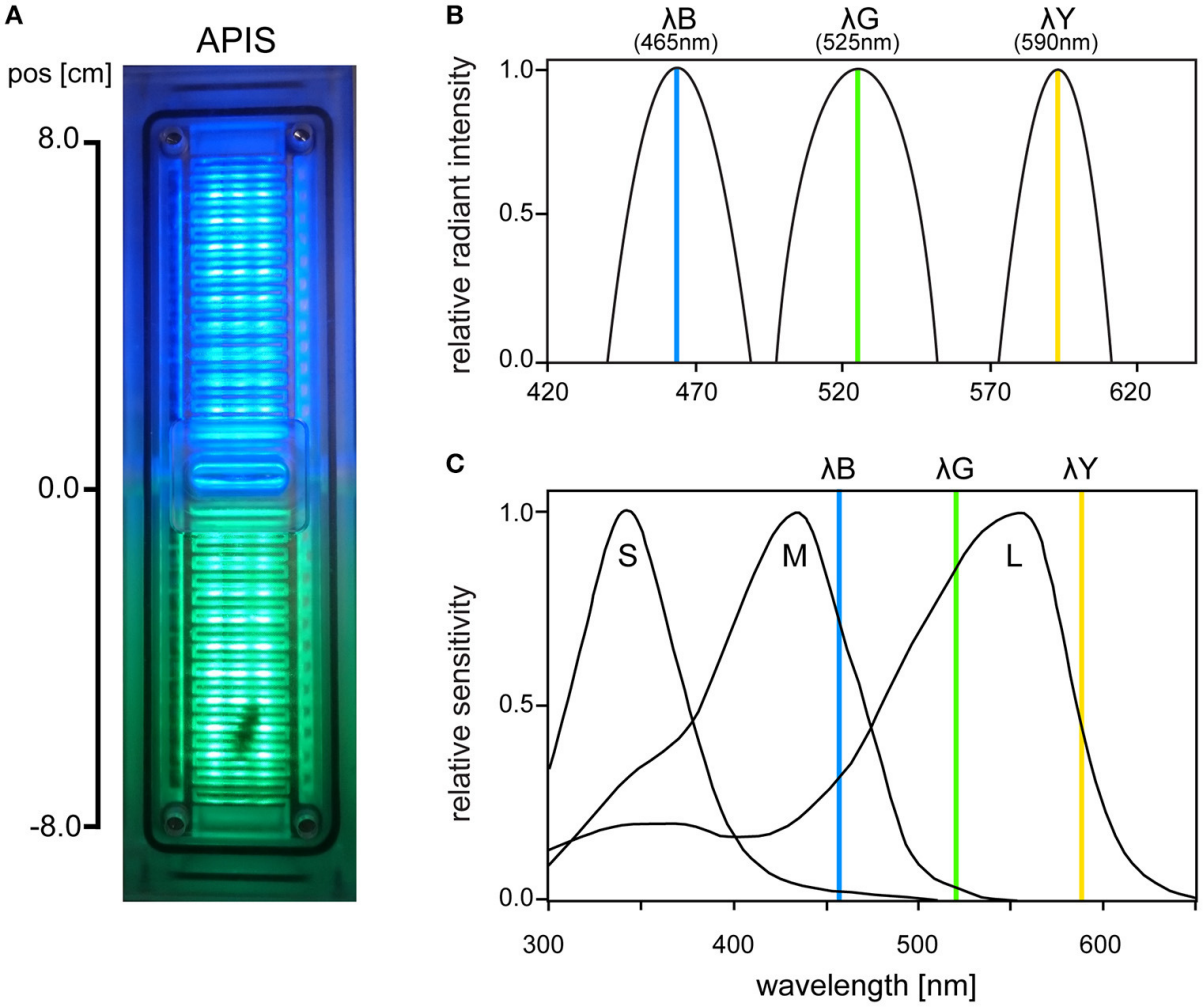
**(A)** Photo of the APIS conditioning chamber from above with bee visible on the lower half. **(B)** Radiant intensity of the three LEDs used in APIS (λ^B^, λ^G^, λ^*Y*^), normalized for each spectrum. **(C)** Spectral sensitivity of the three honeybee photoreceptor types (short, medium, and long wavelength sensitive, respectively). Vertical lines indicate the peak wavelengths of the three LEDs that were used in our experiments (465, 525, and 590 nm, respectively). λ^B^ (465 nm) activated weakly S-, strongly M-, and moderately L-receptors. λ^G^ (525 nm) activated weakly M- and strongly L-receptors, while λ^Y^ (590 nm) moderately activated L-receptors only (short, medium, and long wavelength-activated receptors, respectively). Spectral sensitivity curves are adapted from Hempel De Ibarra et al. (2014) (originally from Menzel et al., 1986).

The APIS conditioning chamber is an aversive conditioning device already established in olfactory conditioning (Kirkerud et al., 2013; Schott et al., 2015; Wehmann et al., 2015). The conditioning chamber consisted of acrylic glass in which a bee can move freely on ceiling and floor (Figure 1A). The bee's movement along the chamber was tracked by 26 infrared LED sensors and opposing phototransistors (16 Hz sampling rate; QEE113 & QSE113/114, Fairchild semiconductor). Interior surfaces were covered by an electrifiable metallic grid and three different colored LED light arrays arranged between the slits of the grid [HCL-1908ABD-4, “super blue“, λ^B^ = 465 nm, Δλ = 45 nm, Luminous Intensity (LI) = 105 mcd; HCL-1903AGC-4, “super pure green,” λ^G^ = 525 nm, Δλ = 45 nm, *LI* = 310 mcd; HCL-1904HY-4, “super yellow,” λ^Y^ = 590 nm, Δλ = 25 nm, *LI* = 330 mcd; Huey Jann Electronics]. The wavelengths of the LEDs were within the spectral sensitivity range of honeybees activating different types and amounts of photoreceptors (Figures 1B,C). The different devices in the chamber (metallic grid, LED sensors, etc.) were connected over a microcontroller SMT board (Savvy128, chip45) and an I^2^C bus controlled 16-channel LED driver (powered by 12 V/1A; TLC59116, Texas Instruments) to a computer via USB. Bees could be conditioned in an automated and interactive manner: The metallic grid and the LEDs were controlled via a custom-written computer software which enabled automatic stimulation by uploading a protocol script. The protocol scripts used in this study utilized interactive feedback to execute commands based on the bee's current position. The bee's movement was logged together with the executed commands and used for offline analysis.

### General parameter settings

Suction trough the center and the ends of the chamber via a vacuum pump was set to 900 mbar to avoid accumulation of alarm pheromones without compromising the bee's movement. No light was presented in the chamber between stimulation trials: Ambient light was shielded by a custom-fit card-board chassis covering the chambers. Chambers were washed frequently first with hot water and then with 70% ethanol. In total eight identical chambers were used for all experiments. In all experiments two different wavelengths were paired by co-activating two optically separate light fields on each half of the chamber (Figure 1A). Previous work on olfactory learning and pilot experiments with lights revealing the speed and reaction/decision times of the bees laid the basis of the stimuli dynamics. The stimulus duration was 14 s and the inter-stimulus intervals was set to 44 s (from light onset to light onset). In each of the specific protocols we presented λ^+^ to the chamber side the bee was in (bee side). In the other half we presented λ^−^ or no light (darkness). The onset side of the light depended on the bee's position in the chamber for each trial, thus, place learning could be ruled out. In reinforced paradigms, electric shock pulses of 10 V with 100 ms duration at 4 Hz were initiated 3 s after light onset on the λ^+^ side. Shock lasted 11 s (total: 44 pulses) and shared the offset with the light stimulus. We identified exhausted bees by means of their walking speed: bees with an average speed of lower than 2 cm/s for the test trials were discarded from the analysis (Figure S1). All learning experiments consisted of a differential aversive conditioning protocol. The training phase had nine identical trials with one reinforced and one safe chamber side (λ^+^ with shocks and λ^−^ without, respectively) if not indicated differently. This was always followed by an immediate test phase without shocks to assess short-term memory.

### Learning light stimuli as signals for danger and safety in aversive conditioning (experiment I)

**Figure 2:**
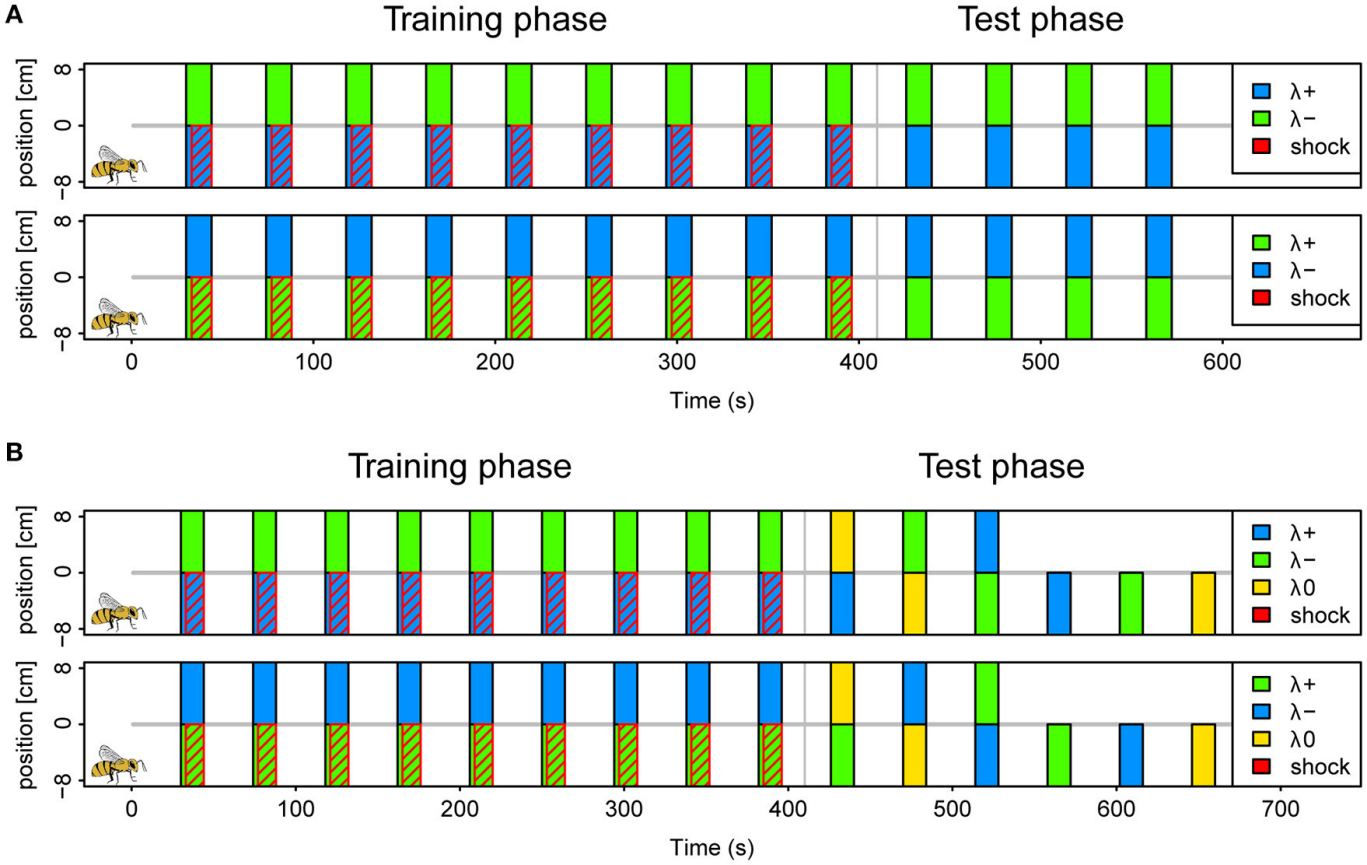
**Protocols for operant aversive conditioning of colored lights in APIS**. **(A)** Schematics of (2 of the 6) protocols used in the reinforced paradigm of experiment I. Trials were distributed at intervals of 44 s (onset to onset). During each trial two colors (λ^+^ and λ^−^) were presented simultaneously on separate halves by LED's below the floor of the chamber for a 14 s period. In the training phase, which consisted of 9 trials, electroshock pulses trailed the color onset by 3 s, and were restricted to the side of the λ^+^ (indicated by red diagonal lines). The bee side is indicated by a bee drawing for the negative positions in the scheme for clarification, but this alternated depending on the bee's position at the respective trial onset. Short-term memory was tested directly after the training in 4 trials without shocks. Again, the λ^+^ was presented on the current bee side, and the λ^−^ on the opposite side. Protocols of three color pairs were used for different bees; blue-green, yellow-blue, and green-yellow with either color of each pair serving as λ^+^ and λ^−^ in a balanced design (top vs. bottom in figure). **(B)** Schematics of protocols used in the reinforced paradigm of experiment II. Training phase was similar to experiment I. During the test phase, 6 different trials were presented with combinations of blue (λ^+^), green (λ^−^) and yellow (λ0) (top panel). These were designed to investigate: avoidance of λ^+^, approach to λ^−^, staying with λ^−^, and overriding of innate phototaxis. The bees received either the three single color trials first, or the three dual color trials. The sequence within each of these triplets was pseudorandomized. Reciprocal protocols with green as λ^+^, blue as λ^−^ and yellow as λ0 (neutral) was used for half of the bees (bottom panel).

We conditioned a total of 405 bees in six different protocols as described above (example protocol illustrated in Figure 2A). Approximately half of the bees were trained in a reinforced paradigm (*n* = 201), whereas the other half was tested for light preferences only (*n* = 204). A total of 30 bees (7.5%) was excluded following the exclusion criterion described above. The protocols correspond to the six possible light combinations of the 3 LEDs (each serving as λ^+^ or λ^−^) at maximal light intensity. The test phase consisted of four unreinforced trials in the corresponding color combination.

### Learning the good or the bad light (experiment II)

We conditioned a total of 279 bees, of which 34 (12%) were excluded following the exclusion criterion described above. Of the total, 159 bees followed the reinforced paradigm and 120 the unreinforced. Conditioning was conducted as in experiment I, with the blue and the green light (λ^B^ and λ^G^) resulting in two protocols where each light served as reinforced or safe, respectively. To investigate what bees learned about the light stimuli the test phase consisted of six pseudorandomized trials with two trials testing the conditioned lights against an untrained third light (λ^Y^), one trial with the λ^−^ (safe light) on the bee side and λ^+^ (shocked light) on the opposite side and 3 trials testing each of the three lights (λ^B^, λ^G^, and λ^Y^) presented on the bee side against darkness on the opposite side (Protocol illustrated in Figure 2B). Hence, the test phase addressed the aversion of the reinforced light, attraction of the safe light (test against λ^Y^ or darkness), passive avoidance of the reinforced light (presentation of λ^+^ on opposite side) and whether learned avoidance could overcome innate phototaxis or whether bees generalized between lights.

### Effects of intensity adjustments on light preferences (experiment III)

A total of 103 bees was tested, of which 5 (4.8%) were excluded. To determine whether bees were biased by the light intensities of the three different LEDs an extensive preference test was performed by varying the intensity of one LED type paired with another LED kept at constant 100% intensity as reference. All possible combinations were tested resulting in six different protocols. The intensity of the variable test light either decreased in the first and increased in the second series (0–100% by 10% increments) or the other way around. Additionally, each stimulus was presented twice, with the reference light on the bee side, and then on the opposite side, in a reciprocal fashion.

### Learning the chromaticity (experiment IV)

We conditioned a total of 99 bees in four protocols consisting of the two color pairs λ^B^λ^G^ and λ^Y^λ^G^. Relative light intensity levels without initial preference bias obtained from experiment III were used. The protocols were similar to the ones described in experiment I, with the exception that instead of an unreinforced group a single unreinforced test was included just prior to the conditioning to assess the bee's innate light preference. A total of 4 bees (4%) was excluded following the exclusion criterion described above.

### Data analysis

We analyzed the APIS-logdata with a custom-written script in R (R-Core-Team, 2012). To capture the different aspects of the bees' behavioral response and evaluate their performances we used three variables: Preference Index (*PI*), Shocks received, and Speed.

*PI* was an assessment of the relative time spent on the safe side during the full response of each trial. It was calculated by subtracting time spent on the λ^−^ side (t_λ−_) with time spent on opposing λ^+^ side (t_λ+_) and dividing on total trial time: *PI* = (t_λ−_–t_λ+_)/(t_λ−_ + t_λ+_), thus ranging from −1 to 1, similar to *Performance Index* from e.g., (Brembs and Wiener, 2006). Subsequently, the *PI* data were fitted with a linear mixed model (lme function in R nlme package, Pinheiro et al., 2012) with trial, light configuration, and paradigm (Reinforced or Unreinforced) as fixed effects, and bee as random effect to account for repeated measurements.

Number of shocks received served as a direct indicator of learning performance for the training phase. Since unreinforced bees in the learning experiments did not receive any actual shocks we estimated the number of fictive shocks that they would receive based on their movement during the 3–14 s (relative to trial onset) if the shocks had been available. In doing this, we could quantify how well-reinforced bees learned to avoid shocks relative to behavior elicited by the light stimuli alone. Number of received shocks in reinforced and unreinforced (actual or fictive, respectively) was fitted in a generalized linear mixed model with a Poisson error distribution where trial, light configuration and paradigm served as fixed effects (predictor variables), and bee identity as random effect (function lmer in the R lme4 package, Bates et al., 2013).

Bees react to electric shocks of 1–12 V by increasing their speed (Figure S2). To assess whether they also increased their speed to light stimuli predicting shocks, and to look separately at the immediate part of the response to the lights without the influence of the shocks, we calculated the absolute speed (distance covered in cm per second) for both the 3 s period just previous (pre) and posterior (post) to light-onset in each trial. The pre-speed was necessary to evaluate whether eventual changes in the post-speed were due to a response to the light itself, or merely a hangover effect from the shocks of the previous trial. Consequently, the speed would constitute the reflex-dominated part of the response. To evaluate whether this reflex was strengthened by the conditioning, we calculated the difference in speed from the first training trial to the first test trial (henceforth referred to as ΔSpeed). One-sample *t*-tests were used to evaluate whether ΔSpeed (in reinforced or unreinforced) was different from 0. Additionally, we fitted post-speed data from all trials in a linear mixed model to assess the effect over trials and between reinforced and unreinforced paradigm. Finally, we evaluated differences in speed for the pre- and post-periods over trials for each protocol. Goodness of fit for residuals of each of the fitted outcome variables was assessed by qq-plots to justify for model selection.

## Results

Honeybees' ability to associate monochromatic lights of different wavelength and intensity with danger or safety was explored in an operant walking assay. We conceded four different experiments where the bees' preference to pairwise presented colored light fields covering each half of the conditioning chamber was tested either in the presence or absence of reinforcement (electric shocks) combined with one of the light fields. The unreinforced group of bees included in the two first experiments was necessary to establish their preference to the different lights and to determine whether they change their preferences over repeated exposures. Hence, it served as a reference for quantification of learning in the reinforced group.

**Figure 3:**
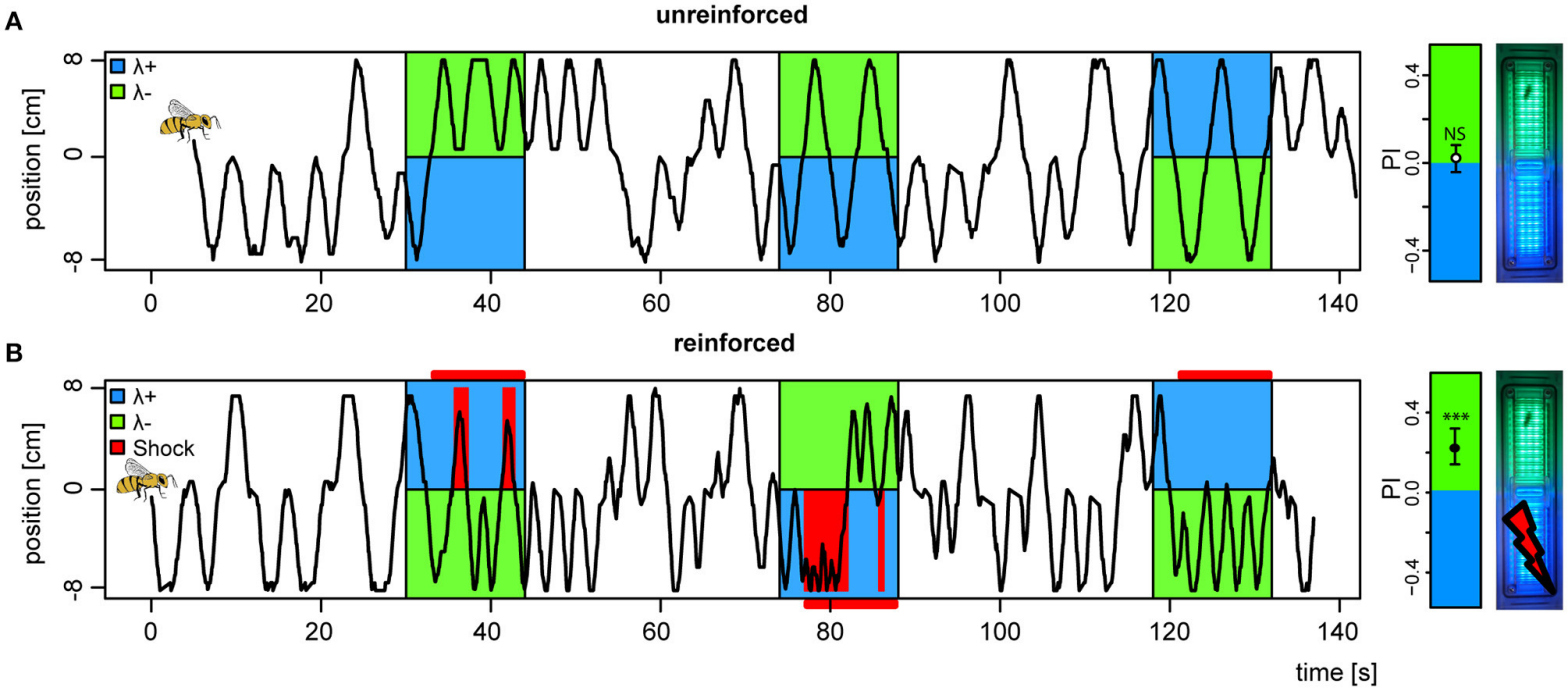
**Change in preference to colored lights following aversive conditioning**. **(A)** Example of positional trace from the first 3 trials of a bee subjected to an unreinforced paradigm, **(B)** and a second bee subjected to a reinforced paradigm (shocks available 3 s after light onset, indicated by horizontal red bars, received shocks as red vertical bars). (far right) Mean ± 95%CI of Preference Index averaged over the four test trials (without shocks) for bees in both unreinforced and reinforced paradigms (n_reinf_. = 36, n_unreinf_. = 32), and photo of APIS chamber. ^***^p < 0.001 and NS, not significant, in a *t*-test against 0.

Following introduction to the conditioning chamber, bees typically explored the dark chamber shuttling back and forth in search of an exit. During light stimuli, unreinforced bees would continue their explorative behavior but with slight biases to either side depending on the light pair they were presented with (example trace, Figure 3A). Reinforced bees on the other hand, abstained from the explorative behavior and stayed away from the shock-paired light (example trace, Figure 3B). Shocks started 3 s after light onset and were restricted to the bee side at light onset, and lasted until the offset of the trial. To completely avoid shocks, the bee would need to escape to the safe light side within the 3 s, and not return until light offset. Hence, our learning paradigm contained an operant element, since bees had to learn from the consequences of their own behavior.

### Learning light stimuli as signals for danger and safety in aversive conditioning

With the first experiment, we investigated whether walking bees could learn to differentiate between two spatially separated but simultaneously presented light fields signaling either danger or safety. All six combinations of the three lights (λ^B^, λ^G^, and λ^Y^) were tested on different groups of bees. An unreinforced group (without shocks) ran in parallel for each light-pair combination.

**Figure 4:**
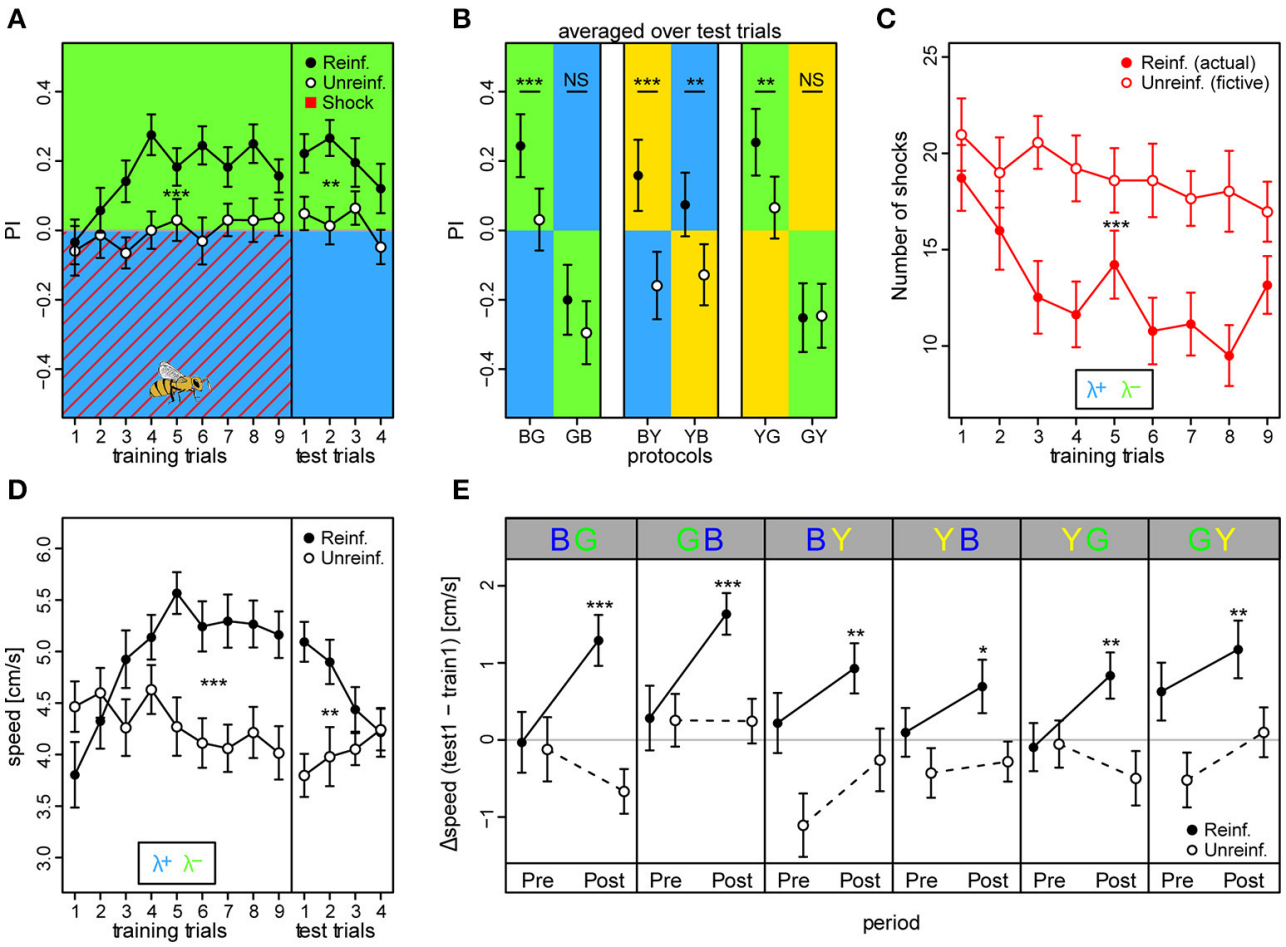
**Walking bees learned to discriminate between two different lights in aversive operant conditioning**. **(A)** Reinforced bees avoided the shock associated light (indicated by red diagonal lines) within few trials, whereas unreinforced bees maintained their initial light preference (*n*_reinf._ = 36, *n*_unreinf._ = 32). Drawing of bee indicates the bee side at stimulus onset relative to the two light fields in all trials (Preference Index of training phase for bees in the remaining 5 color configurations can be found in Figure S9). **(B)** PI averaged over test trials in the 6 conditioning protocols with the reciprocal combinations of all three lights showed that reinforced bees shifted their preference to the safe light compared to unreinforced bees in the four protocols where λ^G^ was not acting as the λ^+^. **(C)** Reinforced bees reduced exposure to shocks over trials, while unreinforced bees would have received a consistently high nr. of shocks had they been activated (fictive shocks plotted). **(D)** Bees increased their speed to light alone (prior to shock onset) over training trials in reinforced compared to unreinforced bees, but decreased over test trials. **(E)** Speed increase from first training trial to first test trial (ΔSpeed) was evident for reinforced bees in each of the six protocols, and occurred directly after light onset (0–3 s **Post** period), but not in the corresponding period just previous to light onset (**Pre**). Thus, indicating that the bees learned to predict danger upon light onset in each of the 6 protocols. Unreinforced bees did not display ΔSpeed >0 for either the **Pre** or **Post** stimulus period. Mean ± SEM of ΔSpeed plotted. Asterisks indicate levels of significance following one-sample *t*-test. Mean ± SEM of data was plotted in (**A,C,D)**, while mean ± 95%CI of data was plotted in **(B)**. Significant differences between reinforced and unreinforced groups (linear regression models in **A–D**) indicated by asterisks (^***^p < 0.001; ^**^p < 0.01; ^*^p < 0.05). Sample size varied between 28 and 36 bees for each of the 6 protocols and 2 treatment groups.

We found that reinforced bees reached higher overall preferences to the safe light during the training phase compared to unreinforced bees (*PI*_reinf_ = 0.16 ± 0.02 vs. *PI*_unreinf._ = −0.01 ± 0.02, mean ± SE over training trials) [protocol BG: *t*_(66)_ = 3.93, *p* < 0.001] (Figure 4A). Consequently, the reinforced bees reduced the number of shocks they received throughout the training phase by ~49% from 18.7 ± 1.7 to 9.5 ± 1.6, while unreinforced bees would have received a constant and higher amount of shocks over trials had they been activated (fictive shocks from 21 ± 1.9 to 18 ± 2.1; actual vs. fictive shocks in BG: *z* = −4.42, *p* < 0.001; Figure 4C). Learning occurred rapidly: reinforced bees displayed efficient avoidance of the shock-paired light within three trials. After nine training trials, a robust short-term memory had formed, reflected in a continued elevated preference to the safe light (*PI*_reinf._ = 0.24 ± 0.05) compared to in unreinforced bees [*PI*_unreinf._ = 0.03 ± 0.05; protocol BG: *t*_(66)_ = 3.42, *p* = 0.001] which was retrievable for four consecutive test trials without apparent extinction [*t*_(203)_ = −1.95, *p* = 0.052].

We trained bees in all six light combinations of the three LED types at their maximum intensity values (see Figures S3–S8 for example traces, and Figure S9 for summary over training and test period). In addition to in the BG protocol, reinforced bees showed an increased preference to the safer light compared to unreinforced bees in the test phase of BY [*PI*_reinf._ = 0.16 ± 0.05 vs. *PI*_unreinf._ = −0.15 ± 0.05; *t*_(53)_ = 3.61, *p* < 0.001], YB [*PI*_reinf._ = 0.07 ± 0.05 vs. *PI*_unreinf._ = −0.13 ± 0.04; *t*_(66)_ = 3.99, *p* < 0.001], and the YG protocol [*PI*_reinf._ = 0.25 ± 0.05 vs. *PI*_unreinf._ = 0.07 ± 0.05; *t*_(62)_ = 3.52, *p* < 0.001] (Figure 4B). Strikingly, a preference change was not evident when λ^G^ served as the shock-paired light: protocols GB [*PI*_*reinf*._ = −0.20 ± 0.05 vs. *PI*_unreinf._ = −0.29 ± 0.05; *t*_(58)_ = 1.026, *p* = 0.31] and GY [*PI*_reinf._ = −0.25 ± 0.05 vs. *PI*_unreinf._ = −0.25 ± 0.05; *t*_(58)_ = −1.044, *p* = 0.30]. PI was higher than zero for test trials in reinforced bees in protocols BG (*t* = 5.43, *p* < 0.001), BY (*t* = 3.05, *p* = 0.0026), and YG (*t* = 5.23, *p* < 0.001), similar to zero for YB (*t* = 1.60, *p* = 0.11), and lower than zero for protocols GB (*t* = −3.92, *p* < 0.001) and GY (*t* = −5.01, *p* < 0.001). This indicated that bees chose the safe side in a non-random way when blue and yellow, but not green light served as λ^+^.

We found that the reinforced bees reacted by increasing their speed over training trials to the light onset alone (averaged over first 3 s following light onset) by ~37% from 3.8 ± 0.3 to 5.2 ± 0.2 cm/s in bees trained in the BG protocol, while the corresponding unreinforced bees kept a near constant speed (from 4.5 ± 0.2 to 4.1 ± 0.2 cm/s; Figure 4D). Indeed, speed increase in training trials was larger for reinforced than unreinforced bees in all six protocols [BG: *t*_(66)_ = 3.49, *p* < 0.001; GB: *t*_(58)_ = 2.98, *p* = 0.0043; BY: *t*_(53)_ = 2.28, *p* = 0.027; YB: *t*_(66)_ = 4.11, *p* < 0.001; YG: *t*_(62)_ = 3.66, *p* < 0.001; GY: *t*_(58)_ = 3.54, *p* < 0.001], suggesting that bees modified their behavior even when λ^G^ served as the shock-paired light. The speed of the reinforced bees also increased over training trials in the dark pre periods (3 s previous to light onset), indicating a general activity increase due to the presence of shocks, but this increase was weaker compared to the post period for all protocols save the GB and GY (Figure S10). For the unreinforced bees, no difference in speed following light onset was apparent (Figure S11). When considering the change in speed from the first training trial to the first test trial (ΔSpeed), reinforced bees in all six protocols displayed a positive speed change of 31.1 ± 4.5% on average in the post light onset period, while in the pre light onset period the speed change was negligible (5.1 ± 3% on average; Figure 4E). For the unreinforced bees, positive ΔSpeed was not observed in any of the protocols. Taken together, these results indicated that the bees anticipated the shocks upon light presentation.

When evaluated over all trials, unreinforced bees showed a slight but significant preference to λ^G^ over both λ^B^ (*PI* = 0.012 ± 0.006) and λ^Y^ (*PI* = 0.014 ± 0.005), when λ^G^ was presented on the bee side [GB: *t*_(372)_ = −2.01, *p* = 0.045; GY: *t*_(360)_ = −2.76, *p* = 0.0061]. This raised the question of whether the observed difficulties in learning to avoid λ^G^ for the reinforced bees was due to the stronger phototactic drive toward this light. We addressed this issue in our fourth and last experiment.

In summary, our results from the first experiment revealed that bees trained in an aversive paradigm change their responses to light stimuli in an adaptive way to avoid electric shocks. Since the λ^+^ and λ^−^ were always presented together, we could not resolve to which extent bees learned to avoid the former, or approach the latter. We also could not tell whether bees would still display the preference shifts when the conditioned lights are presented with darkness on the opposite side of the chamber, thereby opposing phototaxis. These issues were addressed in our second experiment.

### Learning the good or the bad light

In the second experiment, we focused on the BG and GB protocols from the previous experiment, and tested the trained lights λ^B^ and λ^G^ against darkness and against the untrained light λ^Y^ to investigate the extent of which the bees learned to avoid the shock-paired light or approach the safe light. As in the first experiment a reinforced and an unreinforced group of bees were trained to either light configuration.

**Figure 5:**
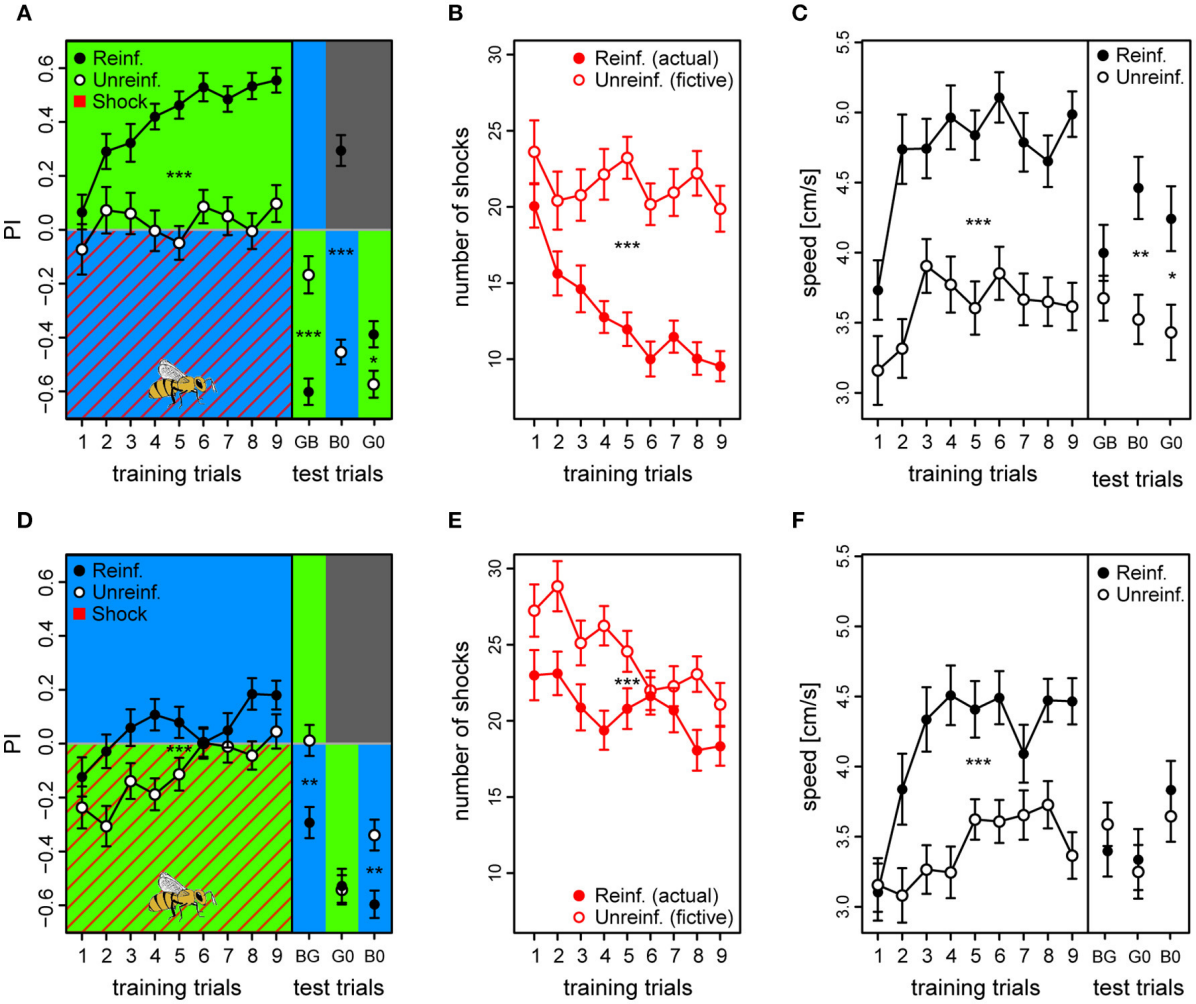
**Acquired valences of conditioned lights are asymmetric for λ^B^ and λ^G^ as λ^+^ (top and bottom row, respectively)**. **(A,D)** Preference Index for each of the training trials of the two reciprocal protocols (BG & GB, respectively), and of the following test trials where λ^−^ was presented on the bee side with λ^+^ on the opposite side (passive avoidance test), and then both lights were presented against darkness. Drawing of bee indicates the bee side at stimulus onset relative to the two light fields for all trials. **(B,E)** Received shocks (actual shocks in reinforced, fictive shocks in unreinforced) for the BG and the GB protocol, respectively. **(C,F)** Speed for the first 3 s following trial onset increased after the first training trials and remained high until the test phase in both protocols. For unreinforced bees the speed increased at a slower rate, and was lower than for reinforced bees. Reinforced bees in the BG protocol reacted with a higher speed than unreinforced also during the test trials (except for trial λ^*G*^λ^*B*^). In the test phase of the GB protocol, reinforced bees only reacted faster to the light onset than the unreinforced for trial λ^G^0 (λ^G^ vs. darkness). Sample sizes ranged from 58 to 65 for each of the four treatment groups. Each variable represented by mean ± SEM. Significant differences between reinforced and unreinforced groups (linear regression models) indicated by asterisks (^***^p < 0.001; ^**^p < 0.01; ^*^ *p* < 0.05).

We found that similar to the first experiment, the reinforced bees of the BG protocol learned to avoid the shocked λ^B^ within few trials and shifted their preference to the safe λ^G^ to a stronger extent than unreinforced bees [*PI*_reinf._ = 0.41 ± 0.02 vs. *PI*_unreinf._ = 0.03 ± 0.02; *t*_(121)_ = 8.05, *p* < 0.001] (Figure 5A; see example trace in Figure S12). Consequently, they received fewer shocks over trials (12.9 ± 1.2) compared to fictive shocks in the unreinforced bees (21.5 ± 1.6; *z* = −19.56, *p* < 0.001; Figure 5B). In the test phase, the reinforced bees preferred the safe λ^G^ [*PI*_reinf._ = −0.61 ± 0.05; GB tested against zero: *t*_(320)_ = −10.68, *p* < 0.001] and even darkness over the previously shocked λ^B^ [*PI*_reinf._ = 0.30 ± 0.06; B0: *t*_(320)_ = 5.18, *p* < 0.001], and they did so to a greater extent than the unreinforced bees [*PI*_unreinf._ = −0.18 ± 0.07 and *PI*_unreinf._ = −0.45 ± 0.05, for the GB and B0 trial, respectively; unpaired *t*-tests, trial GB: *t*_(105)_ = −5.13, *p* < 0.001; trial B0: *t*_(118)_ = 10.2, *p* < 0.001] (Figure 5A). Reinforced bees also preferred the untrained light λ^Y^ over λ^B^ [*PI*_reinf._ = 0.41 ± 0.06; tested against zero: *t*_(320)_ = 7.15, *p* < 0.001] to a greater extent than did the corresponding unreinforced bees [*PI*_unreinf._ = −0.10 ± 0.06; reinf. vs. unreinf: *t*_(120)_ = 5.96, *p* < 0.001] (data not shown). This clearly indicated that the λ^B^ acquired a strong negative valence that even overruled phototaxis. However, both the reinforced and the unreinforced bees preferred the λ^G^ over the untrained λ^Y^ without any apparent difference [*PI*_reinf._ = 0.09 ± 0.07 vs. *PI*_unreinf._ = 0.16 ± 0.06; trial YG: *t*_(121)_ = −0.77, *p* = 0.44] (data not shown). Thus, the data indicated that the bees generalized λ^*Y*^, probably due to its proximity in perceptual space to λ^G^, and that it could not be considered as a perceptually neutral stimulus. For this reason, test trials including the untrained λ^Y^ were omitted from the plot, and we could not conclude that the safe λ^G^ light had acquired an increased positive valence in the BG protocol.

Reinforced bees reacted with a ~30% higher speed than unreinforced in the 3 s period following light onset throughout the training phase (Speed_reinf._ = 4.7 ± 0.2 vs. Speed_unreinf._ = 3.6 ± 0.2) and also during the test trials (except for test trial GB), suggesting that they anticipated the shocks whenever surrounded by λ^B^, while reducing their speed when surrounded by the safe λ^G^ [*t*_(121)_ = 6.39, *p* < 0.001] (Figure 5C). Reinforced bees clearly accelerated upon light onset, which resulted in a significantly higher post onset speed than pre onset speed after the initial training trials [Speed_pre_ = 4.3 ± 0.2 vs. Speed_post_ = 4.7 ± 0.2; *t*_(1, 103)_ = −5.18, *p* < 0.001] (Figure S13A). The unreinforced bees on the other hand, reduced their speed upon light onset throughout the training trials from 4.1 ± 0.2 to 3.6 ± 0.2 cm/s (averaged over trials) [*t*_(984)_ = 5.89, *p* < 0.001] (Figure S13B). This strongly indicated that the bees were able to predict shocks, after only a few conditioning trials.

As for the GB protocol, reinforced bees had an initial preference to the shock-paired λ^G^ (first trial *PI* = −0.12 ± 0.07) which slowly changed toward the safe λ^B^ side over trials (last trial *PI* = 0.18 ± 0.05). However, the preference shift was weaker than for bees trained with the opposite light configuration [*t*_(123)_ = −3.71, *p* < 0.001], and *PI* considered over all training trials of the reinforced bees was only marginally positive [*PI* = 0.057 ± 0.025; *t*_(120)_ = 2.24, *p* = 0.027] despite being higher than the *PI* of the unreinforced bees [*PI*_unreinf_. = −0.11 ± 0.02; *t*_(120)_ = 4.67, *p* < 0.001] (Figure 5D).

The bees trained in the GB protocol were significantly worse at avoiding shocks than the bees trained in the reciprocal protocol (*z* = 9.83, *p* < 0.001). Whereas, bees trained in the reciprocal BG protocol on average reduced the number of received shocks by 52.5% (from 20 to 9.5), GB bees reached a reduction of only 20.4% (from 23 to 18.3) from the first to the last training trial (Figure 5E). Indeed, the bees only reduced shocks slightly more than accounted for by innate preference and/or prior experience, which was reflected by the small but significant difference between average number of shocks in reinforced vs. number of fictive shocks in unreinforced bees (20.7 ± 1.4 vs. 24.5 ± 1.4, respectively; *z* = −4.54, *p* < 0.001; Figure 5E).

In the test phase, reinforced bees did not show any indication of having learned to avoid λ^G^, and clearly spent more time with this light which had just previously been shock-paired instead of crossing over to the dark side as was the case with the opposite protocol [trial G0, PI_reinf._ = −0.53 ± 0.06; tested against zero: *t*_(295)_ = −8.59, *p* < 0.001]. They did so to the same extent as unreinforced bees in the corresponding test trial [PI_unreinf._ = −0.54 ± 0.06; reinf. vs. unreinf. trial G0: *t*_(117)_ = 0.18, *p* = 0.86] (Figure 5D). When λ^G^ was tested against the untrained λ^*Y*^, reinforced bees achieved a negative *PI* of −0.46 ± 0.05 [trial GY tested against zero: *t*_(295)_ = −7.39, *p* < 0.001] (data not shown). In fact, they spent more time with the λ^G^ than the corresponding unreinforced bees did [*PI*_unreinf._ = −0.25 ± 0.07; trial GY: *df* = 116, *t* = −2.33, *p* = 0.021]. This confirmed our findings from the previous experiment, that λ^G^ would not acquire a negative valence under these conditions. On the other hand, reinforced bees did change their attitude toward the safe λ^B^, and showed a preference to this light over the shock-paired λ^G^ [*PI*_reinf._ = −0.29 ± 0.06; BG tested against zero: *t*_(295)_ = −4.77, *p* < 0.001], the untrained λ^Y^ [*PI*_reinf._ = 0.32 ± 0.06; YB: *t*_(295)_ = 5.23, *p* < 0.001], and over darkness [*PI*_reinf._ = −0.60 ± 0.05; B0: *t*_(295)_ = −9.71, *p* < 0.001]. These preferences toward the safe λ^B^ were stronger for the reinforced than the unreinforced bees [BG: *PI*_unreinf._ = 0.01 ± 0.05; YB: *PI*_unreinf._ = 0.09 ± 0.06; B0: *PI*_unreinf._ = −0.34 ± 0.06; PI_reinf._ vs. PI_unreinf._ trial BG: *t*_(120)_ = −3.73, *p* < 0.001; YB: *t*_(119)_ = 2.64, *p* = 0.009; B0: *t*_(119)_ = −3.35, *p* = 0.0011].

Reinforced bees trained with the GB protocol also increased their speed over training trials compared to the unreinforced bees by ~24% [Speed_reinf._ = 4.2 ± 0.2 vs. Speed_unreinf._ = 3.4 ± 0.17; *t*_(120)_ = 4.29, *p* < 0.001], but during the test phase the only clear effect of the reinforcement was an increase in speed to the λ^G^ vs. darkness (G0) trial [*t*_(119)_ = 2.75, *p* = 0.007] (Figure 5F). Similar to in the opposite color configuration, the reinforced bees increased their speed at light onset in anticipation of shocks after a couple of trials [Speed_pre_ = 3.8 ± 0.2 vs. Speed_post_ = 4.2 ± 0.2; *t*_(1, 018)_ = −4.07, *p* < 0.001] (Figure S13C), while the unreinforced bees slowed down at light onset [Speed_pre_ = 4.0 ± 0.2 vs. Speed_post_ = 3.4 ± 0.17; *t*_(1, 052)_ = 8.17, *p* < 0.001] (Figure S13D). Taken together, the results confirmed that bees conditioned in the GB protocol predict the shocks during exposure to λ^G^ without showing an adaptive avoidance behavior lasting through the whole trial as they do when λ^B^ is predicting danger, and suggests that light of this wavelength have a strong innate connotation of safety to foragers.

The results from our second experiment revealed that the valence of either light stimulus (signaling danger or safety) can be modified in the aversive learning paradigm, and that this depends on the light configuration used. Furthermore, learned avoidance can overcome natural phototaxis. Phototaxis was an integral part of our conditioning experiments, and our results from the unreinforced bees in the two first experiments indicated that the bees had slight differences in relative preferences toward the three LED types at their maximum intensities. The role that light intensity played on preference for the different lights was addressed in our third experiment.

### Effects of intensity adjustments on light preferences

In the third experiment, we investigated the bees' preferences for colored light-pairs over varying levels of relative intensities. The goal was to determine if the preferences were intensity-dependent, and whether shifted preferences could be neutralized by adjusting the relative intensities.

Different combinations of a reference light of constant intensity and a test light of variable intensity were tested in absence of reinforcement (example movement trace in Figure S14). Since adaptation in the photoreceptors could potentially be influenced differently to increasing and decreasing intensity increments of lights of different wavelengths, half of the bees received the increasing series first, and the other half the decreasing series first. Each stimulus was presented twice, first with the reference light presented on the bee side, and then on the opposite side, in a reciprocal way. This terminated eventual biases in preference as a result of the bees starting positions at trial onset.

**Figure 6:**
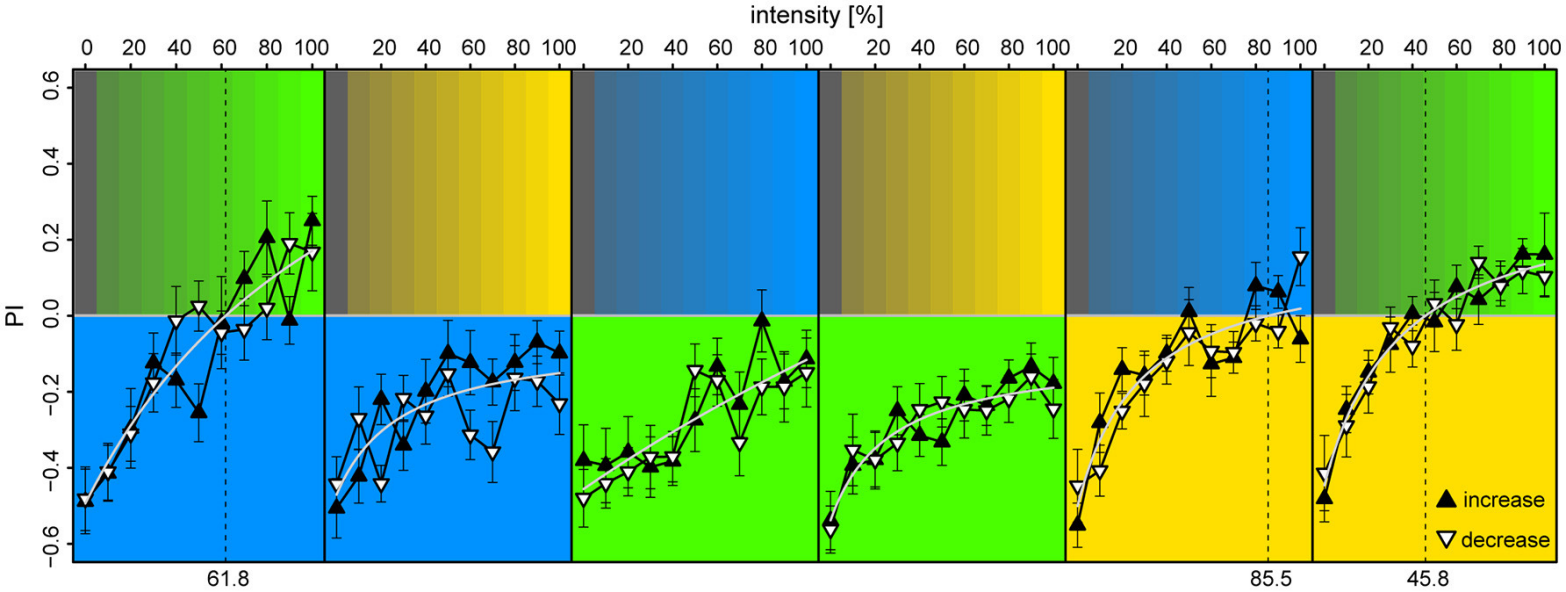
**Preference to colored lights depended on relative intensities**. Each panel corresponds to observed preference in a light pair, in which the lower part indicates the reference light (kept at 100% intensity), while the upper part indicates the test light (varied from 0 to 100% intensity at 10% increments). Each bee was tested with an increasing and a decreasing intensity sequence of a single light pair, where the sequence order was randomized (filled upward triangles and open downward triangles, respectively). To account for starting position bias each stimulus was presented twice: first with the reference light on the bee side, then on the opposite side, and the data points convey pooled averages of the two presentation types. Mean ± SEM plotted, with fitted Michaelis-Menten functions (light-gray curves) for each stimulus pair [*y* = a + bx/(c+x)]. Broken vertical lines indicate estimated zero-preference intensities (x solved for y = 0). *N* = 98 bees (15–18 for each protocol).

We found that the bees displayed strong phototaxis for all three LEDs at 100% intensities vs. 0% (darkness) with no differences in *PI* between the colored lights [*PI*_λB_ = −0.49 ± 0.08, *PI*_λG_ = −0.46 ± 0.09, *PI*_λY_ = −0.52 ± 0.08; Anova: *F*_(2)_ = 0.03, *p* = 0.97] (Figure 6). Relation between *PI* and relative intensities were fitted with a Michaelis-Menten function for each stimulus pair [*y* = a + bx/(c+x), where y is the *PI*, x the light intensity in %, and a,b,c are fitted constants], and levels of zero-preference were estimated by solving x for *y* = 0. With λ^B^ as the reference-light (at 100% intensity), bees preferred λ^G^ for intensities >62%, while λ^B^ was preferred over all tested intensity values of λ^*Y*^. When λ^G^ served as reference, it was preferred over all intensity levels for both λ^B^ and λ^*Y*^. With λ^*Y*^ as the reference, λ^B^ reached zero-preference at 84% and plateaued, while λ^G^ was preferred for intensities >46%. Altogether, these results indicate that λ^G^ was the preferred light over both λ^B^ and λ^Y^, but that this preference could be titered by adjusting intensity levels. The increasing or decreasing series of intensities for the test-light did not result in significant preference differences when evaluated over the full intensity range [*t*-test, BG: *t*_(372)_ = 0.34, *p* = 0.73; GB: *t*_(438)_ = −1.0, *p* = 0.32; BY: *t*_(416)_ = −1.85, *p* = 0.065; YB: *t*_(416)_ = −0.49, *p* = 0.63; YG: *t*_(416)_ = −0.34, *p* = 0.73; GY: *t*_(416)_ = −0.28, *p* = 0.78]. The slight discrepancies between preferences observed in this experiment vs. the ones observed in the unreinforced bees of the first two experiments were expected, due to the test light being presented on the opposite side of the bee at onset in half of the trials.

The results from our third experiment revealed that the bees' preferences to lights of different wavelengths depend on intensity, and that they can be neutralized by adjusting the intensities of the individual lights. This experiment provided us with the intensity values needed to explore learning absent of biases in phototaxis for the different colored lights.

### Learning the chromaticity

In the fourth experiment, we conditioned bees with intensity adjusted light pairs of equal relative preferences obtained from the previous experiment. The four color-configurations of the first experiment that included the λ^G^ (BG, GB, YG, and GY) were used, but instead of unreinforced groups, a single preference test was introduced prior to training (see Figures S15–S18 for example traces). The λ^B^ or λ^*Y*^ lights at 100% intensity was paired with either 62% or 46% of λ^G^ to ensure that there was no initial preference bias for any of the lights (values acquired from experiment III).

**Figure 7:**
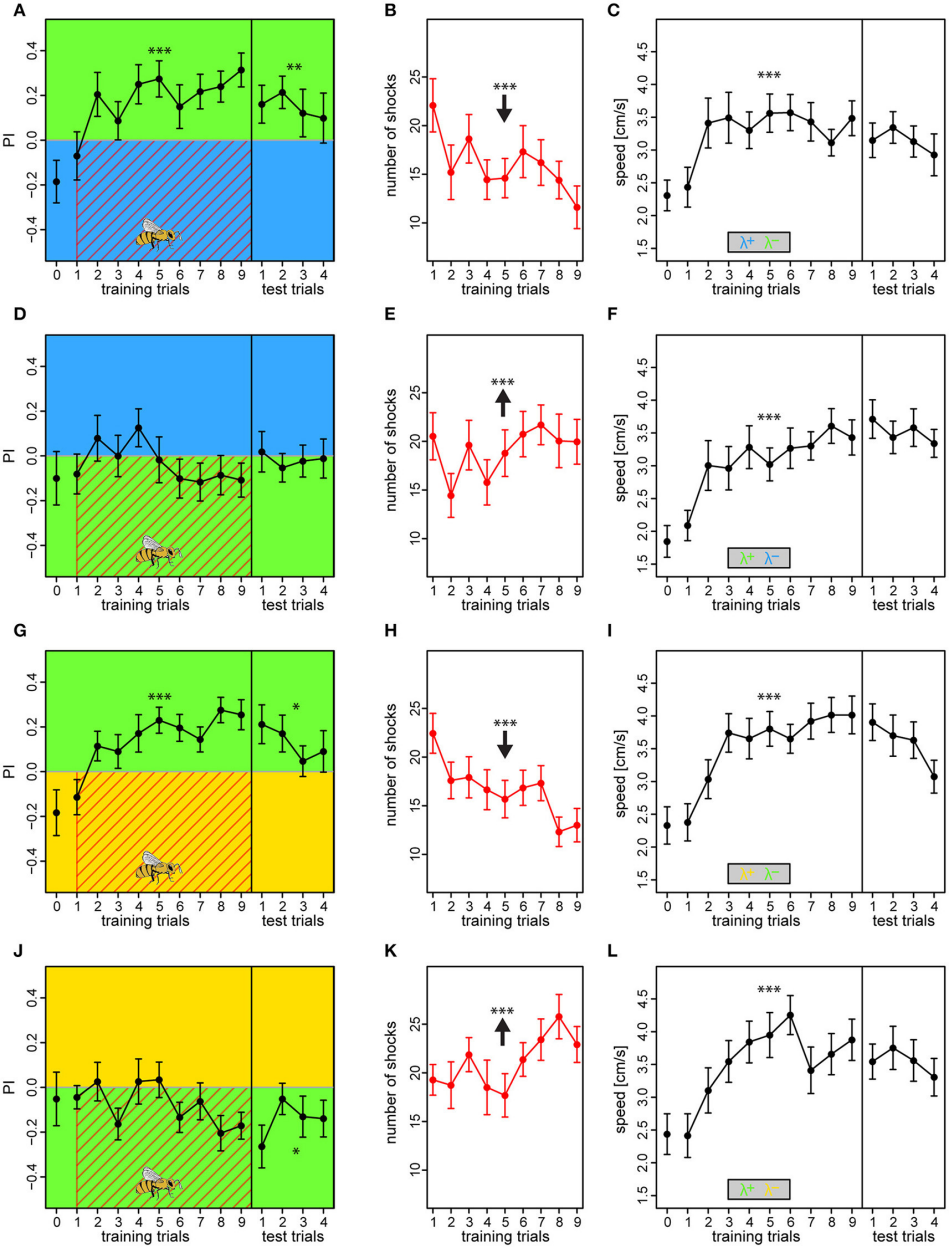
**Compensating biases in light preferences by adjusting light intensities results in similar learning outcomes**. The two light pairs in which asymmetric learning occurred in the previous experiments (λ^B^λ^G^ and λ^Y^λ^G^) was retested with reduced intensities of λ^G^, to 62% when used against λ^B^, and to 46% when used against λ^Y^. Bees received an additional preference test as the very first trial to establish a reference, otherwise, the protocols were similar to experiment I. Bees trained with λ^B^ or λ^Y^ as the reinforced light successfully increased their preference to the safe λ^G^ side over trials **(A,G)**, and thus decreased the nr. of received shocks over trials **(B,H)**. On the contrary, bees trained with λ^G^ overall failed to change their preferences **(D,J)**, and thus received a constant or even increasing nr. of shocks over trials **(E,K)**. Drawing of bee indicates the bee side at stimulus onset relative to the two light fields for all trials. Bees in all protocols displayed anticipative behavior by increased their speed to the light onset alone (averaged over first 3 s) over trials **(C,F,I,L)**. Each variable represented by mean ± SEM. Significant changes over training trials or difference from 0 in test trials (linear regression models) indicated by asterisks (^***^p < 0.001; ^**^p < 0.01; ^*^p < 0.05). *N* = 95 (22–25 bees for each protocol).

Bees conditioned with either λ^B^ or λ^*Y*^ as shock-paired lights and λ^G^ as safe light successfully changed their preference toward the latter [BG: *PI* = 0.18 ± 0.03, *t*_(199)_ = 2.78, *p* = 0.006; YG: *PI* = −0.15 ± 0.02, *t*_(199)_ = 4.12, *p* < 0.001] (Figures 7A,G), and consequently reduced number of received shocks over trials from 22.1 ± 2.7 to 11.6 ± 2.2 and from 22.4 ± 2.0 to 13.0 ± 1.7 shocks for the BG and YG protocol, respectively (BG: *z* = −7.45, *p* < 0.001; YG: *z* = −8.62, *p* < 0.001; Figures 7B,K). This preference was still apparent in the test phase, demonstrating robust short-term memory [tested against zero, BG: *PI* = 0.15 ± 0.05, *t*_(91)_ = 2.97, *p* = 0.0038; YG: *PI* = 0.13 ± 0.04, *t*_(91)_ = 2.59, *p* = 0.011]. On the contrary, bees trained in the reciprocal light configurations with λ^G^ as the shock-paired light did not learn to avoid it, and *PI* was either flat or even decreasing over training trials [GB: *PI* = −0.03 ± 0.03, *t*_(183)_ = −1.76, *p* = 0.08; GY: *PI* = −0.08 ± 0.02, *t*_(175)_ = −2.04, *p* = 0.0439] (Figures 7D,J), and they received an increasing amount of shocks over trials (GB: *z* = 3.36, *p* < 0.001; GY: *z* = 5.49, *p* < 0.001; Figures 7E,K). In the test phase, these bees either showed no preference to either light or preference to the previously shock-paired light for the GB and GY protocols, respectively [GB tested against zero: *PI* = −0.02 ± 0.04, *t*_(91)_ = −0.33, *p* = 0.739; GY: *PI* = −0.15 ± 0.04, *t*_(91)_ = −2.75, *p* = 0.0073]. Bees in all protocols showed anticipative behavior by a ~46–67% speed increase to the light onset alone during the course of training [RM-Anova: *F*_(756)_ = 58.8, *p* < 0.001]. No difference between protocols was found, which justified pooling of the speed data [RM-Anova: *F*_(3, 91)_ = 1.2, *p* = 0.3] (Figures 7C,F,I,L). Bees did not increase speed over training trials in the period previous to light onset [RM-Anova: *F*_(7, 56)_ = 0.16, *p* = 0.68], and again no difference between protocols was evident [RM-Anova: *F*_(3, 91)_ = 0.98, *p* = 0.4]. The outcome was congruent with that of the previous experiments where relative light intensities differed. Hence, we excluded that relative intensity differences affected the learning, and also that bees failed to learn to avoid λ^G^ due to a strong phototactic drive, but rather due to innate or acquired positive meaning of light of this wavelength.

In short, our results from the fourth experiment revealed that bees learn the lights of equal relative preference similarly to when the relative intensities differ. Thus, in the aversive paradigm relative intensity played only a minor role compared to the wavelength of the lights, suggesting that chromaticity is the most salient feature for the bee to utilize.

## Discussion

Despite the honeybee's imperative role in research on learning and memory throughout the last century, the absence of studies on aversive operant learning is striking. In this study, we demonstrated that walking honeybees can learn to use lights of different wavelengths and intensities to predict danger or safety in an aversive discriminatory operant conditioning paradigm.

### Aversive learning of colored lights is asymmetric

We found that avoidance learning was asymmetric for lights of three different wavelengths, even though these wavelengths are known to activate overlapping subsets of photoreceptors. By using these wavelengths, we could confirm that bees trained in an avoidance paradigm could differentiate between colors that are clearly separated in the perceptual space, λ^B^ (465 nm, human blue) and λ^Y^ (590 nm, human yellow) or λ^G^ (525 nm, human green), as well as two colors that are considerably closer, λ^G^ and λ^Y^. Essentially, when either λ^B^ or λ^Y^ predicted shocks, bees learned to effectively avoid these lights within few trials. However, when λ^G^ was shock-paired, avoidance responses remained comparatively unchanged (Figures 4, 5). Even after removing the initial bias in phototaxis by adjusting light intensities, our results revealed that bees still had severe difficulties in learning to avoid λ^G^ (Figure 7). This indicates that bees have an innate representation of safety for green, which is hard to overcome by aversive operant conditioning.

To the best of our knowledge, difficulties in associating green light (or objects) with danger has not been previously shown in bees. Variation in learning rates of different colors has been described in appetitive conditioning of free flying bees, but all tested colors (within the visual range) were eventually learned, and mostly within a handful of training trials (Menzel, 1967). Africanized honeybees were recently shown to display a similar asymmetric color dependent learning in an appetitive PER assay (Jernigan et al., 2014). The bees could readily learn that blue and green light predicted a reward, but failed to do so for violet light. The authors speculated in whether light within the UV range needs to be polarized for the bees to learn it. Indeed, another study demonstrated that bees were able to learn in a discriminatory task when polarized UV light, but not polarized blue or green light was rewarded (Sakura et al., 2012). Under these circumstances, the resistance of green light to acquire a negative valence would be interesting to retest with directionality added to the light. Like us, bees can see more shades of green than of any other color (Giurfa et al., 1996), and it is conceivable that it could be an evolutionary benefit to keep the most abundant color in nature from easily acquiring negative valence. Alternatively, green might be irrelevant for bees as a target color, given that plant leaves are generally the background to flowers, and flowers have evolved colors that are not green. Nature presents colors in various patterns and combinations. Thus, it would be interesting to test bees with combinations of colors to study further asymmetries in aversive learning. For instance, it is conceivable that the occurrence of green together with light of another wavelength could acquire a negative valence. Ultimately, it remains to be elucidated whether this phenomenon is a result of acquired information about green through extensive foraging or of inherent properties of the network responsible for shock evaluation and color processing.

### Honeybees are capable of relief learning

In the second experiment, we disentangled the aversive and the appetitive memory constituents by presenting the danger (λ^+^) and safe-predicting light (λ^−^) against darkness and an untrained light (λ^Y^) in the test phase. We found that honey bees can either form a negative association to the shock-paired or a positive one to the safe light, but not both at once for the light combinations we tested (Figure 5). The increased preference for the safe-predicting light (λ^−^) that we observed argues for honeybees being capable of relief learning, similar to what has been demonstrated in mammals conditioned in avoidance paradigms with discriminatory stimuli (references within Mackintosh, 1974, chapter 6 & 10). In most groups of three conditioned stimuli there will be two that are closer in perceptual space—in our experiments, these are green and yellow light. Since bees learned to differentiate between green and yellow when the latter was shock-paired in experiment I and IV (Figures 4B,G), yellow light was selected as untrained test light with blue and green as either λ^+^ or λ^−^ in experiment II. Our results indicate that bees generalized the green and yellow light. Thus, we could not assume that yellow served as perceptually neutral in this setting. In order to draw conclusions about what was learned about the blue and green lights without the interference of generalization, we opted to emphasize on the test of trained lights against darkness instead. Mackintosh discusses that mammals show learning toward both the S+ and S– in most cases, but that what is learned about S+ and S– varies a great deal depending on the experimental procedures. Having both acquisition processes running in parallel would be more demanding for the nervous system, and thus the most important feature (dependent on context and state of the animal) could be prioritized. In our case, it is conceivable that we only observed that bees learned either λ^+^ or λ^−^ strictly due to their difficulties in assigning valence to the green light. Alternatively, generalization might be stronger for the safe light compared to the dangerous one, causing green and yellow to be treated more similarly than blue and yellow due to the shorter distance in color space. A third conceivable possibility is that the phototactic preference to the green light over yellow (with both lights at max intensity) which was evident in the unreinforced bees, over-shadowed an eventual learned attraction to the safe green light. However, the two latter points would not explain why we did not observe an increased preference to green (when serving as λ^−^) against darkness in reinforced bees compared to unreinforced bees. To pinpoint whether a bee can learn about both the shock-paired and safe light at once, the green light would need to be replaced with a third light (in addition to blue and yellow) which also can acquire a valence. Light in the UV range is an obvious candidate, and should be tested in future experiments.

Learning the valence of the light predicting danger is arguably of higher importance to the animal than learning about the safe light, but acquiring a compound memory for both the danger and the safety signal would be the optimal. Behavioral responses shaped by relief learning appear to be of weaker strength (Tanimoto et al., 2004; Gerber et al., 2014; Vogt et al., 2015), which corresponds well with our own findings. However, it is important to note that our paradigm differed from the previous studies on relief-learning in *Drosophila*, most notably in that it contained an operant element (shocks could be avoided). Classical and operant processes involve partly separate cellular and molecular pathways (Lorenzetti et al., 2006; Ostlund and Balleine, 2007; Brembs and Plendl, 2008). Thus, it is possible that the experienced relief differs as a consequence of this.

Due to its implications in a range of afflictions like addiction, phobia, post-traumatic stress, compulsive disorders and anxiety, interest in the reward-punishment brain circuitry engaged during avoidance conditioning and relief learning is increasing (Krypotos et al., 2015). Since fundamental aspects of valence attribution to conditioned stimuli due to stimulus timing is shared across phyla (Gerber et al., 2014), underlying cellular mechanisms are likely to be related.

Recently, honeybees were shown to exhibit learned helplessness following inescapable and unavoidable punishment (Dinges et al., 2017), a renown and well-studied phenomenon in higher animals considered to be an underlying cause of depression. Interestingly, bees did not show the typical reduction in activity, rather they remained highly active but failed to restrict their movements to a safe location. This behavior corresponded with what we observed for bees trained with green light as the shock-paired stimulus in our avoidance paradigm. However, since the bees in our paradigm still were in control of the shocks, we cannot ascertain whether the exhibited behavior of the bees from the two studies was due to the same underlying mechanisms.

The relative simplicity and accessibility of the honeybee nervous system combined with its sophisticated behavior and cognitive capabilities makes it an ideal model organism, and the paradigm we present here could readily be combined with pharmacological manipulations to further the understanding of the underlying mechanisms of important phenomena like relief learning and learned helplessness.

### Unreinforced bees prefer the brightest light

Since the LEDs we used had different brightness levels at max intensities (λ^B^ = 105 mcd, λ^G^ = 310 mcd, λ^*Y*^ = 330 mcd), and the honeybee photoreceptor types are differently sensitive to these wavelengths, we expected that the bees would show different levels of attraction toward the three colors when presented in pairs. Phototaxis in bees is considered to be independent of chromatic properties of light (Labhart, 1974; Menzel and Greggers, 1985), particularly for wavelengths that activate the M- and L-receptors (medium and long wavelength-activated, respectively). Assuming that the M- and L-receptor types contribute similarly to phototaxis, we would expect our λ^G^ LED to be the most attractive one given its high absolute intensity (310 mcd) and that it activates L-type strongly (relative activation ≈ 0.8, Figure 1C) and M-type weakly (0.05). While the λ^Y^ despite having a high absolute intensity as well (330 mcd) only activates the L-type moderately (0.3), and the λ^B^ despite activating all three receptor types (S-type = 0.05, M-type = 0.7, and L-type = 0.3 relative sensitivity, respectively) had a much weaker absolute intensity level (105 mcd). This corresponds well with our results from the unreinforced light preference experiments, where we observed a preference to the λ^G^ over the other two lights (Figure 6). Interestingly, we found that for the λ^B^λ^Y^ light pair, the λ^B^ preference was comparably stronger when serving as the constant intensity reference light, than when serving as the intensity variable test light. This indicates that the visual system in honeybees is less sensitive to intensity changes in light of long wavelengths (yellow). The fact that the preference bias could be eliminated by reducing the intensity of λ^G^ argued for the chromaticity being of little importance for bees in a phototaxis test. Nonetheless, it is important to point out the distinction between color-ignorance and color-blindness. Clearly, the bees are able to see the difference between lights based on chromaticity, but without the right incentives in the right context, emphasis is shifted toward relative intensities instead.

### Chromaticity trumps intensity of lights during learning

In the two first learning experiments, we used the three LED types at their maximum values. This meant that bees could potentially take advantage of the difference in perceived intensity in addition to the difference in wavelengths to discriminate between the lights. However, our results from these experiments were congruent with results of the fourth experiment in which light intensities were balanced. This implies that preference bias due to intensity had negligible effect on the learning performance, and confirmed that the bees mainly learned the chromaticity of the lights. Other color conditioning experiments with either free flying or harnessed bees where colored lights of equal or different brightness were used, have indicated that bees learn the chromatic properties of the lights before the intensity differences when the visual angle and the chromatic distances are large (Giurfa et al., 1997; Mota et al., 2011b). By increasing the relative intensity differences, while reducing the color distance between the light stimuli, one could assess the point where the two light characteristics are of equal informational value to the bees during aversive conditioning in APIS.

### Bees predicted the shock period

Behavioral responses from the period between light onset and shock onset is of particular interest, since it is dissociated from the reinforcement and contains the part of the response where the initial action selection takes place. Restriction in temporal resolution prohibits assessment of short periods in conventional operant learning assays, but due to the high sampling rate of the IR tracking sensors in APIS, we could isolate and evaluate the responses from this period. By comparing the speed from the 3 s period just previous to the light onset, with the speed from the period 3 s just after the light onset (but before shock onset), we found that bees in the reinforced paradigm increased their speed upon light onset (Figure 4E, Figures S10, S13). This speed increase in the post onset period took place after a couple of training trials, and since it was larger than the speed increase in the corresponding dark pre-period, it indicated that the bees could predict the upcoming shock period. Interestingly, we observed this predictive behavior even in the protocols in which green light was reinforced, and avoidance behavior considered for the full trial period was absent. This indicates that the initial response is most likely to the light onset itself, and appears to be independent on chromaticity.

Prediction of the upcoming shock events has also been revealed in the immediate response of walking bees conditioned with odors in APIS (Kirkerud et al., 2013). There we quantified the directional speed (velocity) in the period between odor and shock onset and revealed that bees increased velocity away from the CS+ but not the CS-. Unlike odors that are injected from the distal ends into the chamber, colored light fields do not provide adequate directional information to the bees. Thus, the most advantageous strategy for the bee upon light onset, is to increase the speed in the current movement direction until arriving at the safe half of the chamber. Since the starting position and movement direction of the bee at light onset was random, any variable that evaluates the immediate response of the bee and depends on the direction or starting position will have a high degree of variance. Therefore, we found non-directional speed to be the most suitable variable to capture US-independent anticipative behavior in our visual learning paradigm.

## Conclusion and outlook

In this study, we demonstrated that honeybees can learn colored lights in an aversive walking paradigm, and found that the information about either the light signaling danger, or the light signaling safety is learned. Thus, demonstrating for the first time, that bees are capable of relief learning in an avoidance conditioning paradigm. They learned to predict shocks following light onset regardless of chromaticity (contextual learning), but they had difficulties in associating green light with danger, and failed to avoid green light even after extensive conditioning. This implied that light of this wavelength had an innate and/or acquired meaning of safety in foragers. The neural substrates underlying visual learning is yet to be unveiled, and our paradigm accompanied with i.e., pharmacological treatments could prove to be a promising approach.

## Author contributions

NK: conceded of and designed the experiments, analyzed the data and wrote the paper. US: assisted in designing experiments, carried them out and assisted in the analysis of the data and in revision of the manuscript. CG: assisted in experimental concepts and designs, guided the analyses and the presentation of the data, and revised the manuscript.

## Funding

This work was supported by funds of the Federal Ministry of Food and Agriculture (BMEL) based on a decision of the Parliament of the Federal Republic of Germany via the Federal Office for Agriculture and Food (BLE) under the innovation support programme. NK participates in the International Max-Planck Research School for Organismal Biology (IMPRS), and funding for US's assistance in the experimental work was partly provided by them.

### Conflict of interest statement

The authors declare that the research was conducted in the absence of any commercial or financial relationships that could be construed as a potential conflict of interest.

## Equation

$$PI=(t_{\lambda -}-t_{\lambda +})/(t_{\lambda -}+t_{\lambda +})$$

## Nomenclature

λ^B^ = 465 nm (human blue, bee blue-green).

λ^G^ = 525 nm (human green, bee bright green).

λ^Y^ = 590 nm (human yellow, bee dim green).

λ^+^ = shock-paired color.

λ^−^ = safe color.

BG = conditioning protocol where blue light is shocked and green light is safe.

GB = green light is shocked and blue light is safe.

BY = blue light is shocked and yellow light is safe.

YB = yellow light is shocked and blue light is safe.

YG = yellow light is shocked and green light is safe.

GY = green light is shocked and yellow light is safe.
