# Supplementary material for: Aversive Learning of Colored Lights in Walking Honeybees
Source: Front Behav Neurosci. 2017 May 22;11:94. doi: 10.3389/fnbeh.2017.00094 (PMC5438982; doi:10.3389/fnbeh.2017.00094)
Supplement: Supplementary file 1 [file DataSheet1.docx]

Supplementary Material

Aversive learning of colored lights in walking honeybees

**Nicholas H. Kirkerud, Ulrike Schlegel, & C. Giovanni Galizia**

*** Correspondence:** Nicholas H. Kirkerud, [nihaki84@gmail.com](mailto:nihaki84@gmail.com)

# Supplementary Figures and Tables


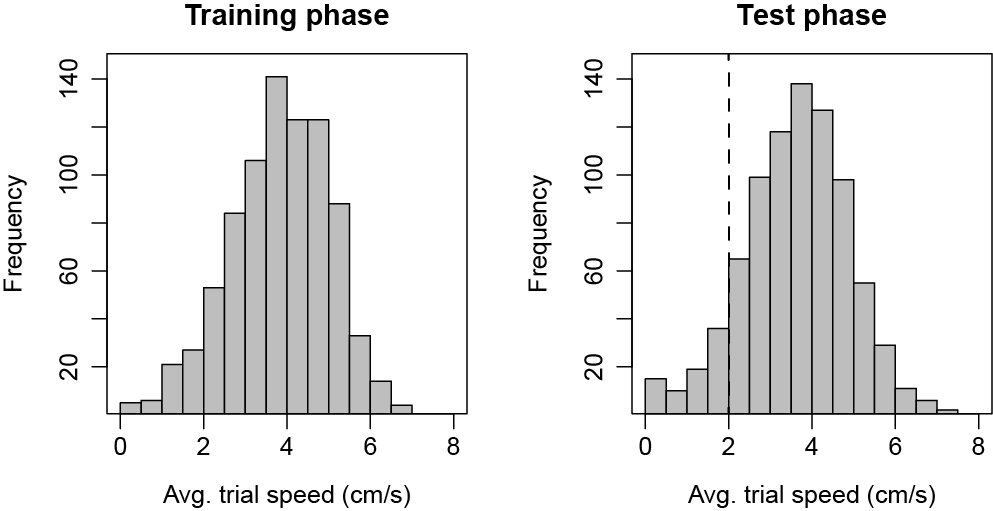


Figure S 1: Distribution of speed averaged over trials from bees in all color learning experiments. Broken line indicates the cutoff at 2cm/s, which was set as the threshold value for inclusion in analysis due to satisfying levels of activity. According to this criterion, 9.7% of bees were excluded.


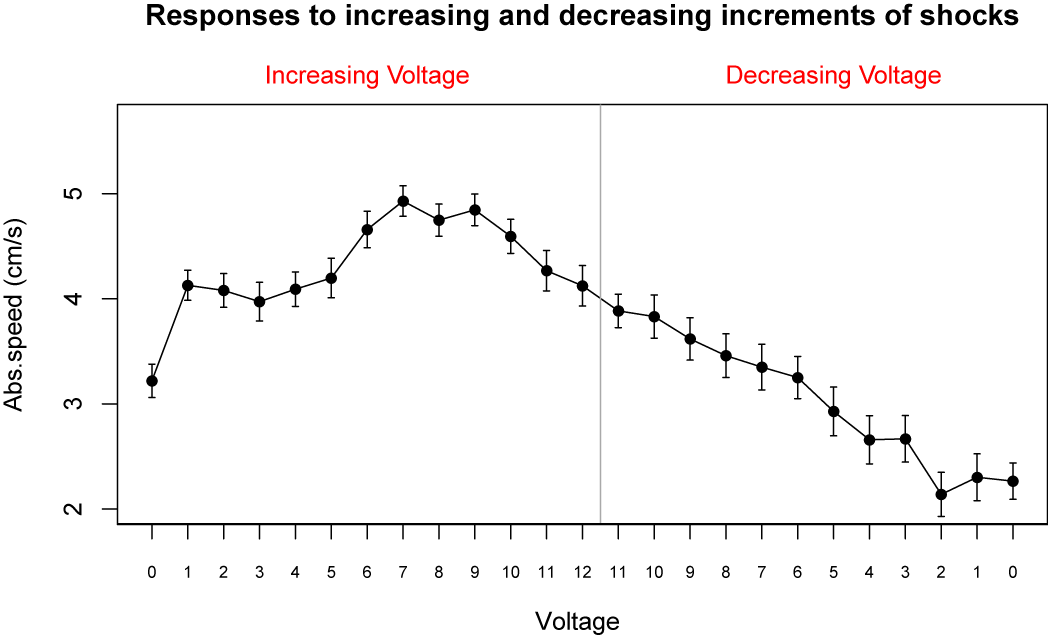


Figure S 2: Absolute speed of bees correlates positively with electric shock. Bees (n = 38) were presented with 23 trials of 6 seconds containing 24 inescapable shock pulses (4 Hz, 100ms duration) with an ITI of 60s. In the first 12 trials, the voltage increased in increments of 1 unit per trial, whereas in the latter 11 trials it decreased by 1 unit per trial. An empty trial (no stimulus) was included as a reference both 60s before the 1st shock trial, and after the last one. The absolute speed was calculated as total distance covered divided on trial duration for each trial as a measure of change in general direction-independent activity.


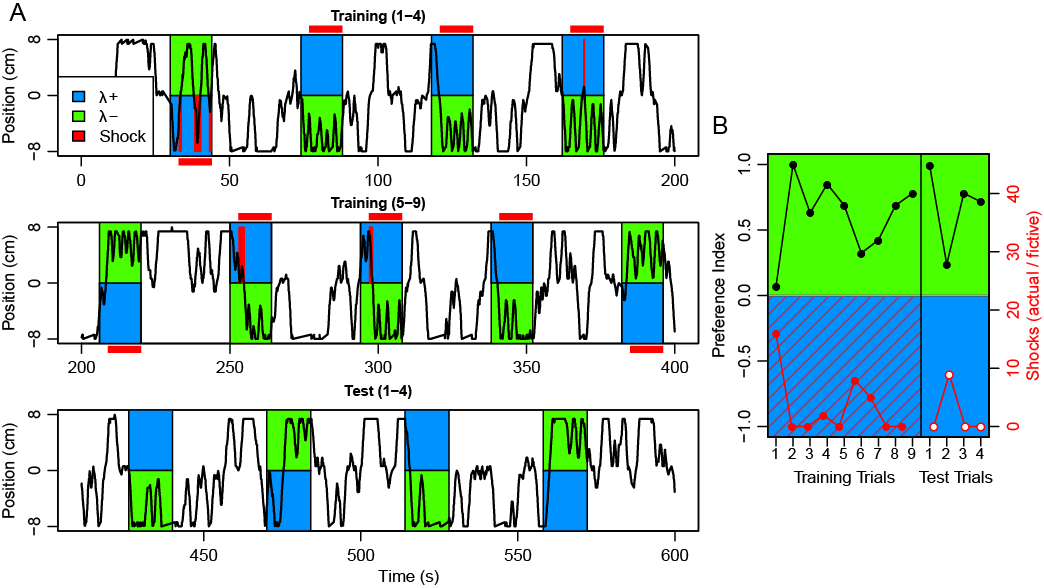


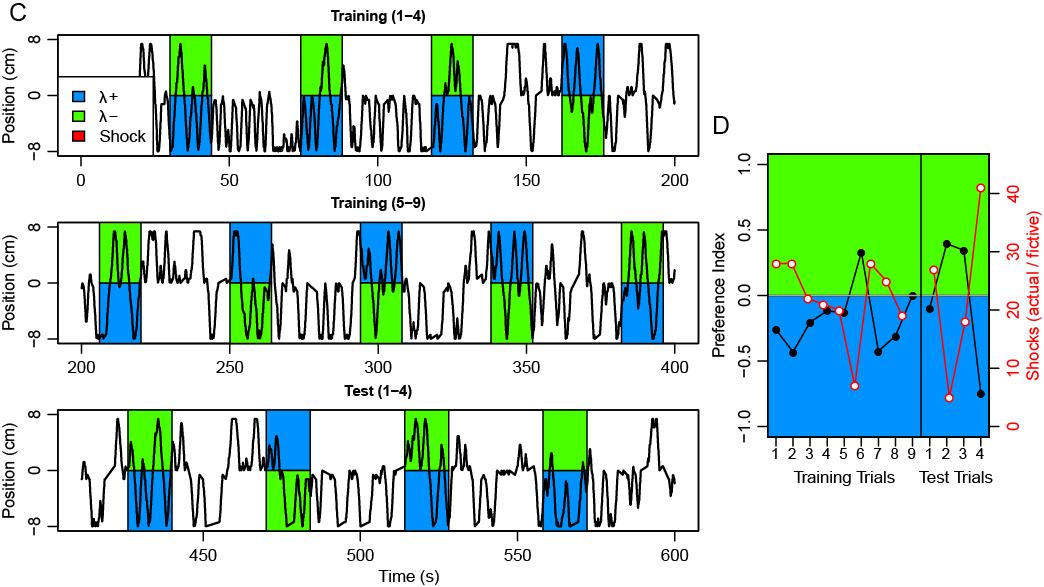


Figure S 3: (A) Example trace of bee conditioned with blue light as reinforced and green light as safe stimulus. (B) Bee successfully discriminated from the 2^nd^ trial on and spend most of the time one the safe side for the remaining training and test trials. Preference Index over trials is plotted in black, while actual shocks (for training trials) and fictive shocks (for test trials) received are plotted in red filled and white filled circles, respectively. (C) Example trace of bee with the same light stimuli configuration but in the unreinforced paradigm (both lights were safe). (D) Bee did not discriminate or show any clear preference towards the two light fields.


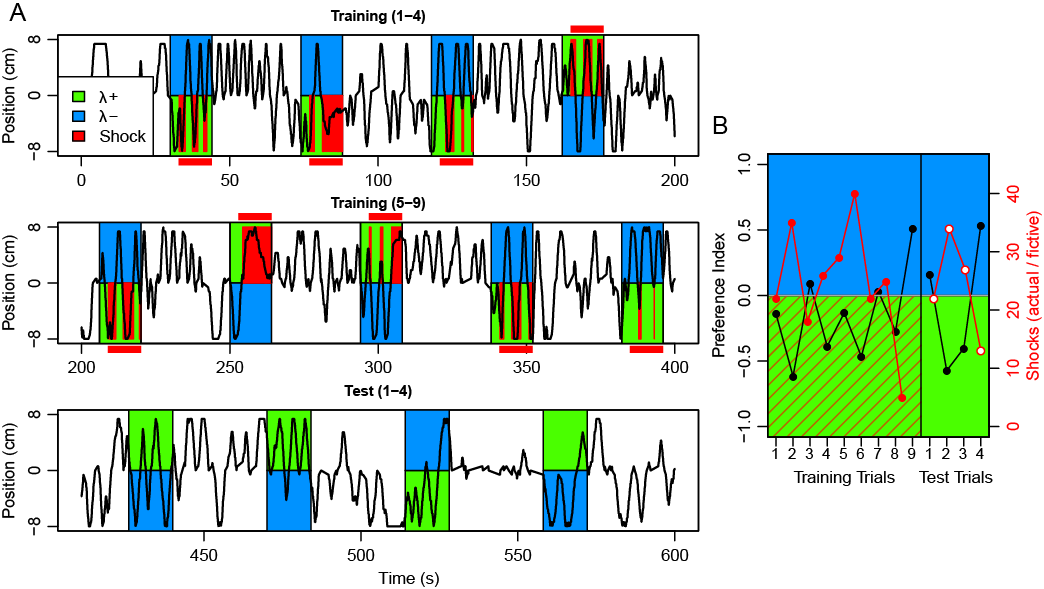


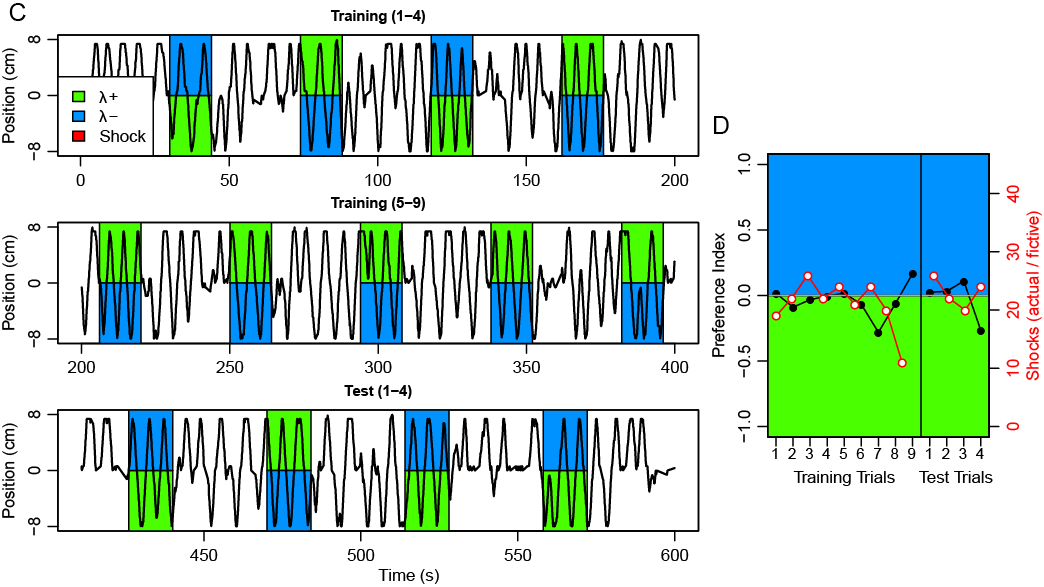


Figure S 4: (A) Example trace of bee conditioned with green light as reinforced and blue as safe stimulus. (B) Bee did not display any clear preference to either colors throughout the experiment. Preference Index over trials is plotted in black, while actual shocks (for training trials) and fictive shocks (for test trials) received are plotted in red filled and white filled circles, respectively. (C,D) Example trace of bee with the same light stimuli configuration but without any reinforcement showed no preference towards either light field.


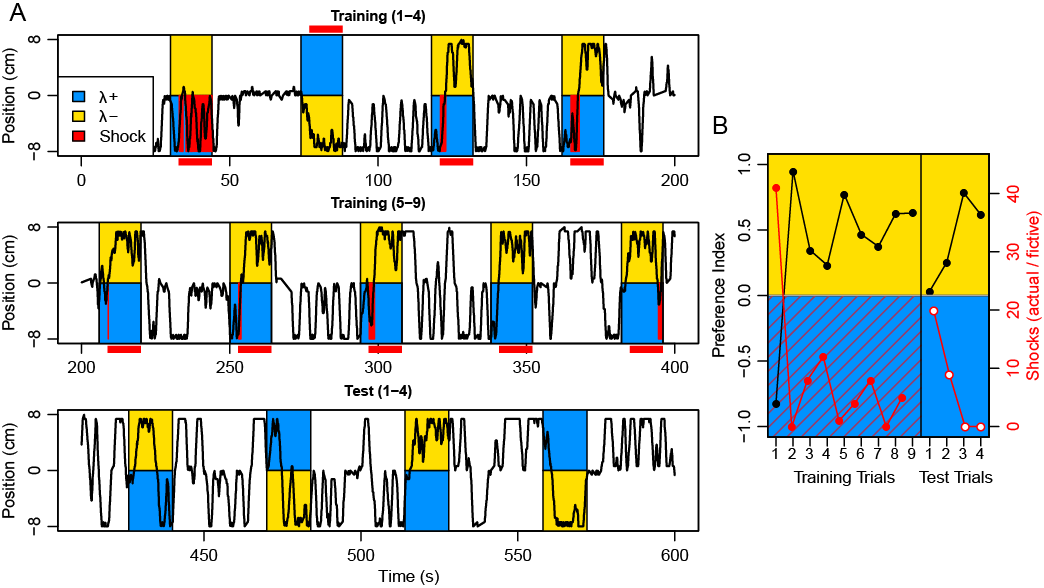


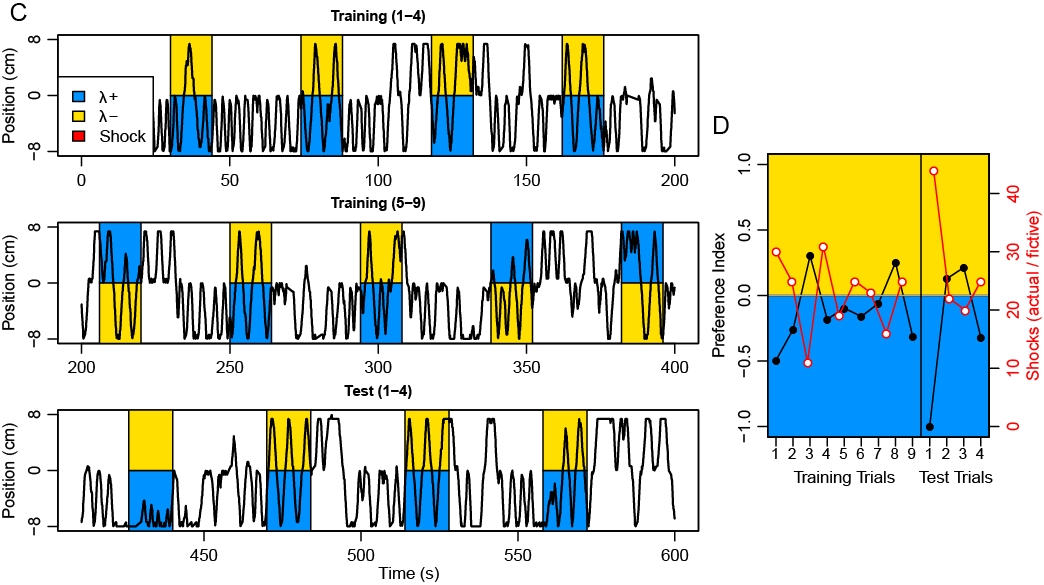


Figure S 5: (A) Example trace of bee conditioned with blue light as reinforced and yellow light as safe stimulus. (B) Bee successfully discriminated from the 2^nd^ trial on and spend most of the time one the safe side for the remaining training and test trials. Preference Index over trials is plotted in black, while actual shocks (for training trials) and fictive shocks (for test trials) received are plotted in red filled and white filled circles, respectively. (C) Example trace of bee with the same light stimuli configuration but in the unreinforced paradigm (both lights were safe). (D) Bee did not discriminate or show any clear preference towards the two light fields.


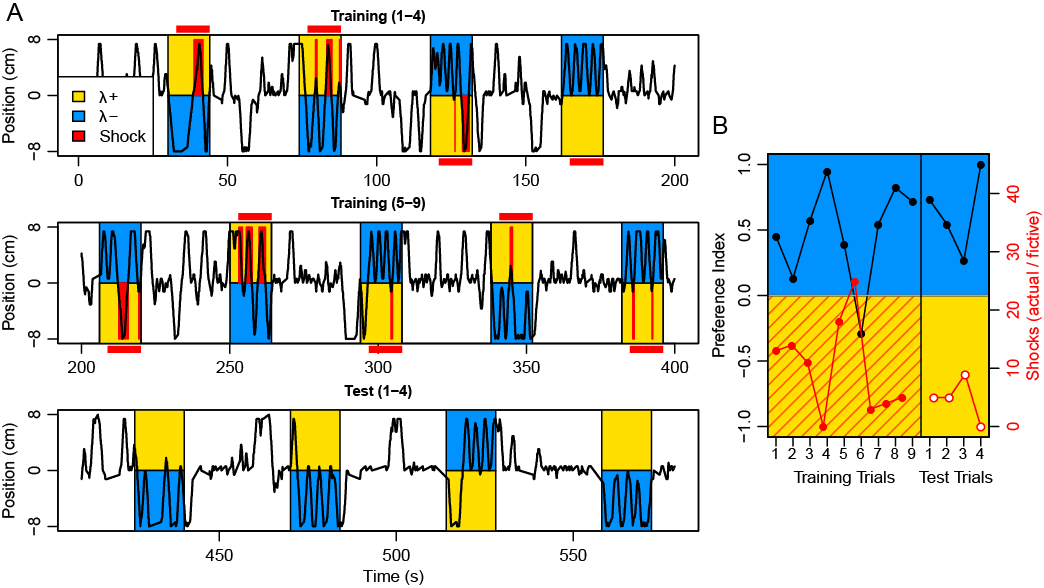


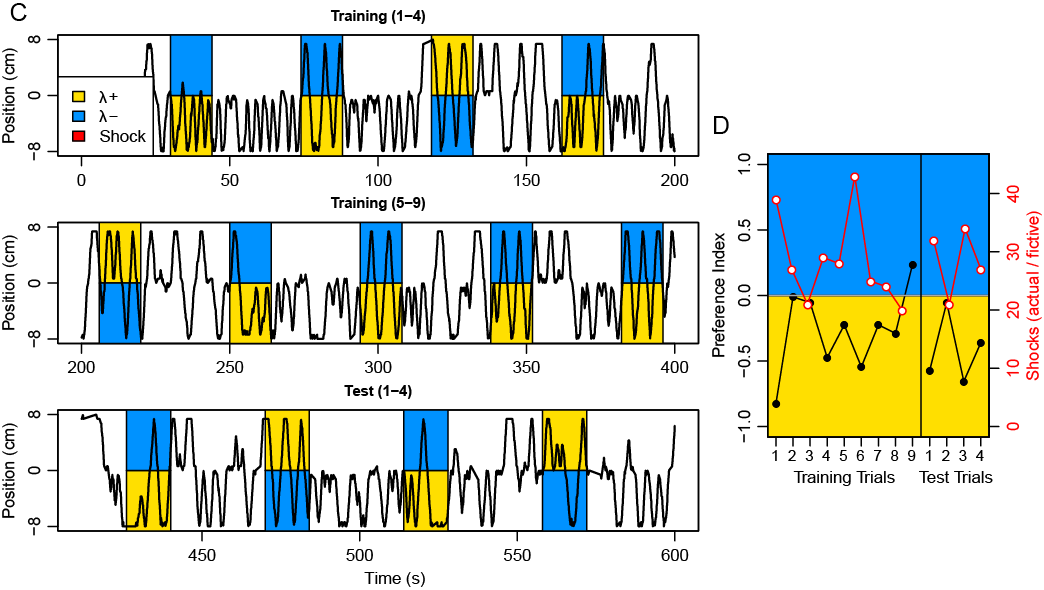


Figure S 6: (A) Example trace of bee conditioned with yellow light as reinforced and blue light as safe stimulus. (B) Bee successfully discriminated from the 2^nd^ trial on and spend most of the time one the safe side for the remaining training and test trials. Preference Index over trials is plotted in black, while actual shocks (for training trials) and fictive shocks (for test trials) received are plotted in red filled and white filled circles, respectively. (C) Example trace of bee with the same light stimuli configuration but in the unreinforced paradigm (both lights were safe). (D) Bee maintained a slight preference to yellow light throughout the experiment.


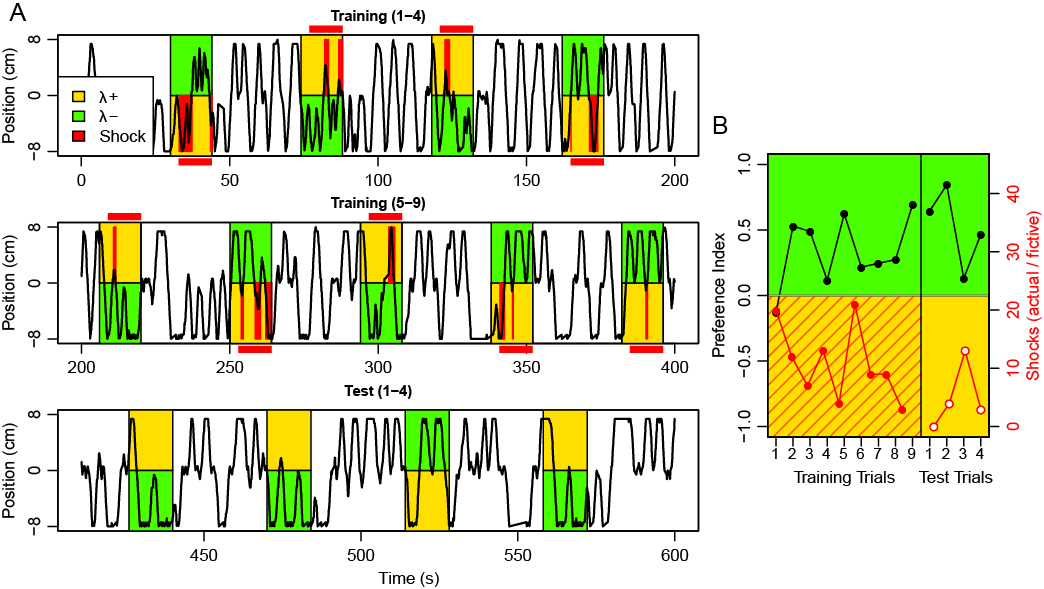


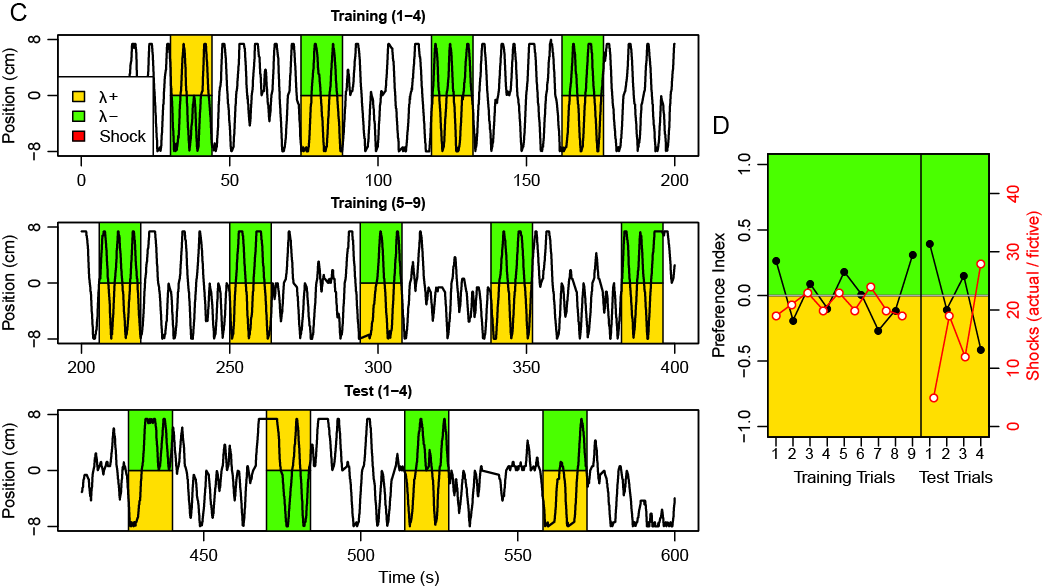


Figure S 7: (A) Example trace of bee conditioned with yellow light as reinforced and green light as safe stimulus. (B) Bee successfully discriminated from the 2^nd^ trial on and spend most of the time one the safe side for the remaining training and test trials. Preference Index over trials is plotted in black, while actual shocks (for training trials) and fictive shocks (for test trials) received are plotted in red filled and white filled circles, respectively. (C) Example trace of bee with the same light stimuli configuration but in the unreinforced paradigm (both lights were safe). (D) Bee did not discriminate or show any clear preference towards the two light fields.


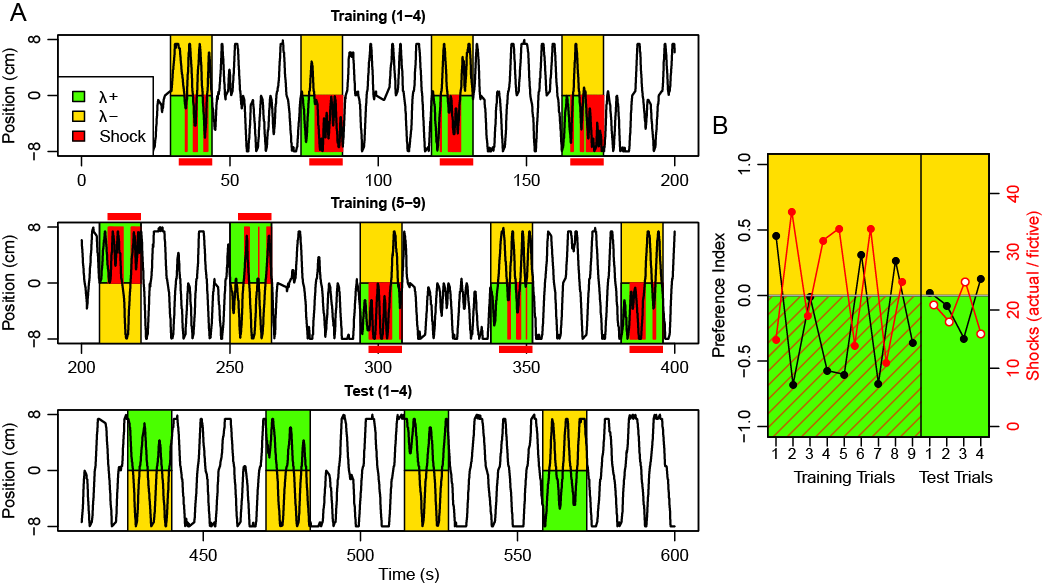


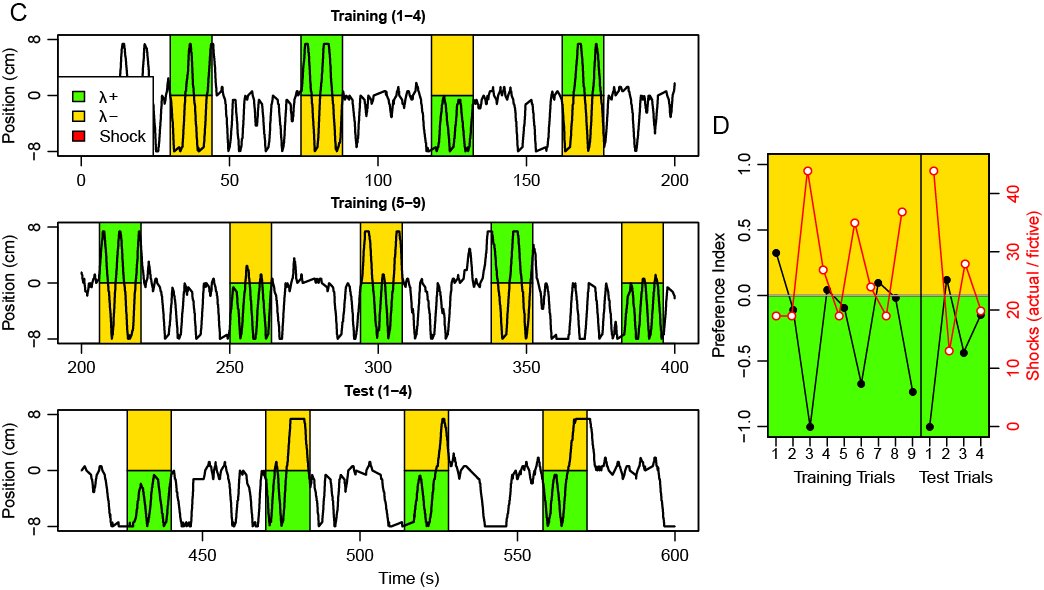


Figure S 8: (A) Example trace of bee conditioned with green light as reinforced and yellow as safe stimulus. (B) Bee did not display any clear preference to either colors throughout the experiment. Preference Index over trials is plotted in black, while actual shocks (for training trials) and fictive shocks (for test trials) received are plotted in red filled and white filled circles, respectively. (C,D) Example trace of bee with the same light stimuli configuration but without any reinforcement showed no preference towards either light field.


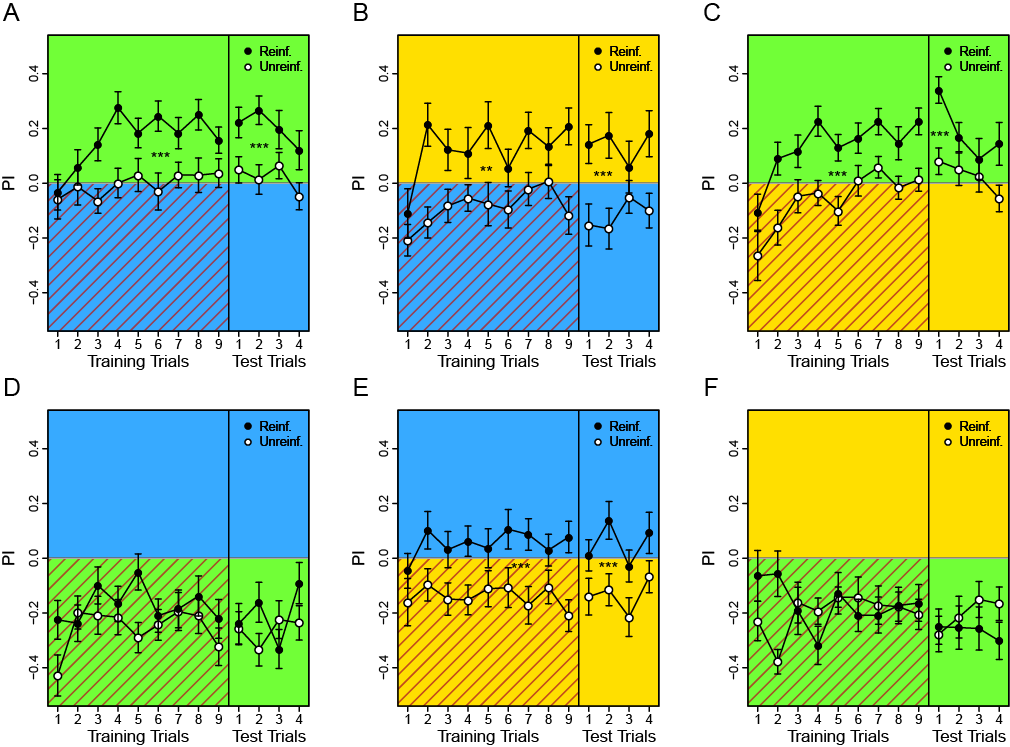


Figure S 9: Preference Index (mean±SE) from reinforced (black) and unreinforced (white) bees for each of the 6 protocols in experiment 1. Note that bees could learn all three colored lights as safe (A,B,C,E), while they had difficulties in learning green light as indication of danger (D and F). Asterisks display significant differences between reinforced and unreinforced bees over the training and test phases obtained by fitting linear regression models. Sample sizes varied from 27-36 bees for the 12 groups (6 protocols x 2 paradigms).


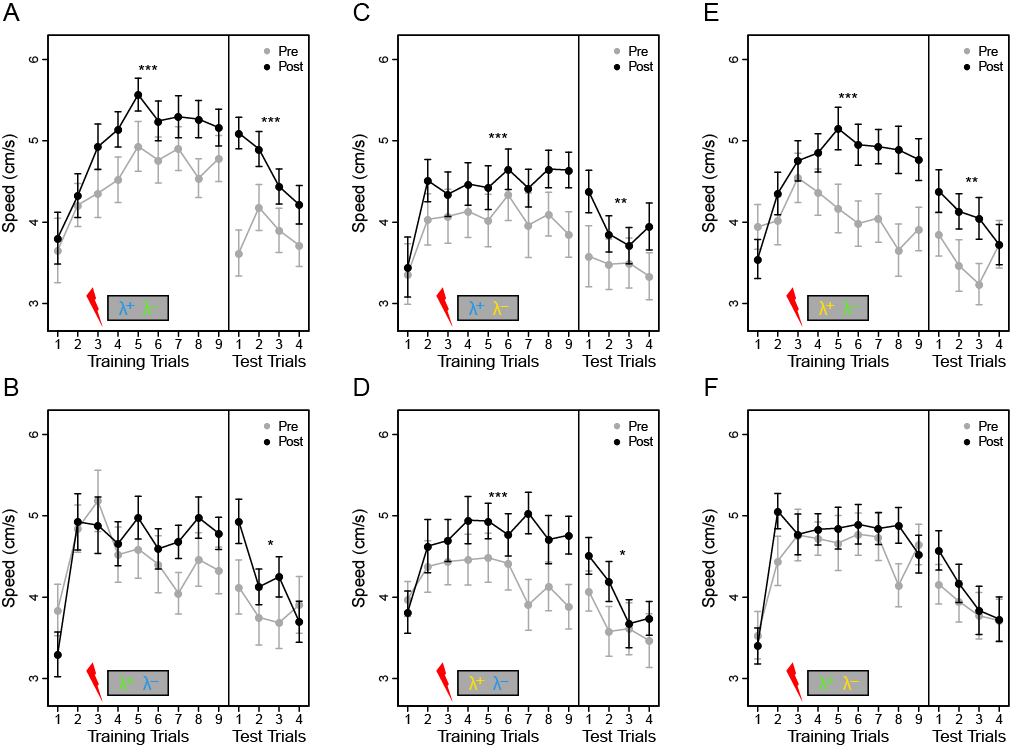


Figure S 10: Bees accelerated in order to avoid incoming shocks after light onset. Speed (mean±SE) of pre vs post light onset period from reinforced bees for each of the 6 protocols in experiment1 (A:BG, B:GB, C:BY, D:YB, E:YG, F:GY). All periods are before shock onset. Bees in the reinforced paradigms responded with higher speed in the post period than in the pre period, and this difference was significant in 4 out of the 6 protocols. The speed response in the reinforced bees went down over test trials, indicating extinction of the initial response. Asterisks display significant differences between pre and post period over the training and test trials obtained by fitting linear regression models. Sample sizes varied from 28-36 bees for the 6 reinforced groups.


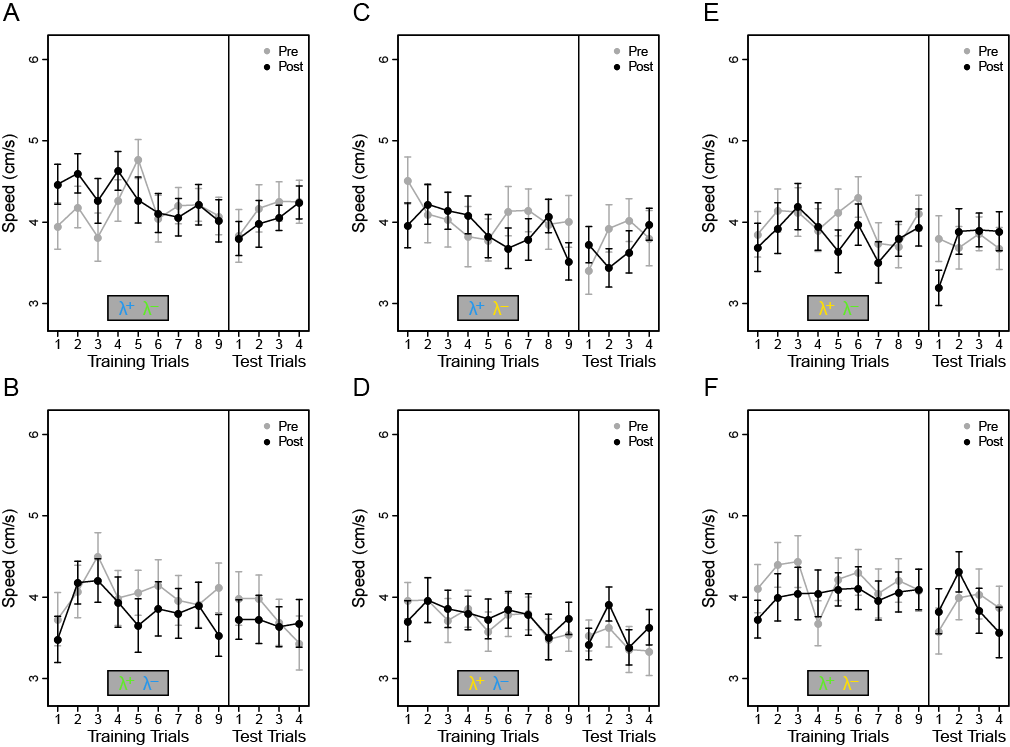


Figure S 11: Light onset alone did not affect speed in unreinforced bees. Speed (mean±SE) of pre vs post light onset period from unreinforced bees for each of the 6 protocols in experiment1 (A:BG, B:GB, C:BY, D:YB, E:YG, F:GY). No difference in speed were apparent in either the training or test phase for any of the six protocols. Sample sizes varied from 27-33 bees for the 6 unreinforced groups.


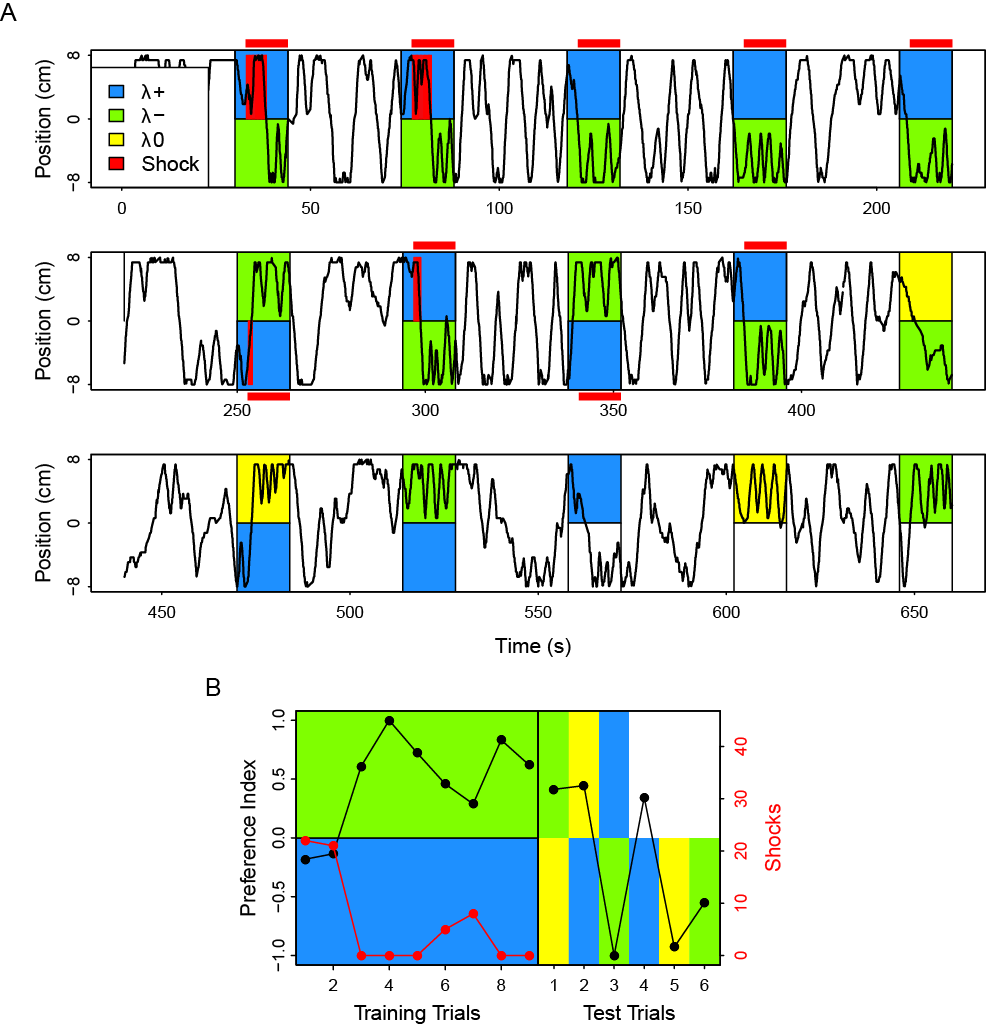


Figure S 12: (A) Example trace of bee conditioned and tested with green light as reinforced, blue as safe, and yellow as neutral stimulus. The protocol consisted of 9 training trials where the side of the blue light field was paired with electroshocks while the side of the green light field was safe. Subsequently, the bee underwent 6 test trials with different combinations of the conditioned lights, a neutral light (yellow), and darkness to investigate the contribution of each color and to test if the learned avoidance could overcome natural phototaxis. (B) Bee quickly changed its preference towards the safe (green) light, and thus avoided most electric shocks throughout the training trials. In the test phase, the bee was attracted to green, and avoided blue when yellow was the alternative. Both yellow and green light fields were preferred over darkness, while the previously reinforced blue light field was not.


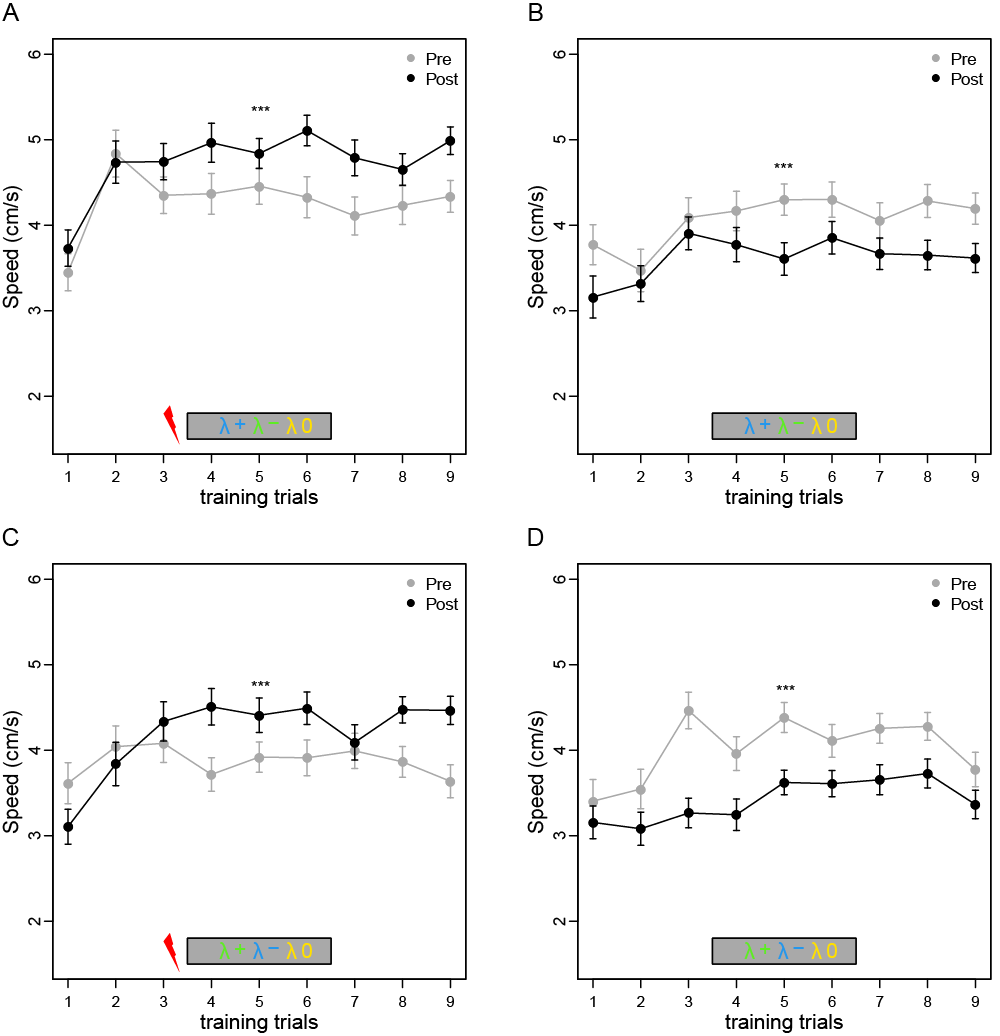


Figure S 13: Bees accelerated in order to avoid incoming shocks after light onset. Bees trained in the reinforced paradigm (A&C) of both the BGY and GBY protocols responded to the light onset by increasing their speed in the post periods compared to in the dark pre periods. Unreinforced bees on the other hand, reduced their speed in the post period (B&D). Sample sizes ranged from 58-65 for each of the four treatment groups. Each variable represented by mean+SEM. Significant differences between pre and post periods (linear regression models) indicated by asterisks.


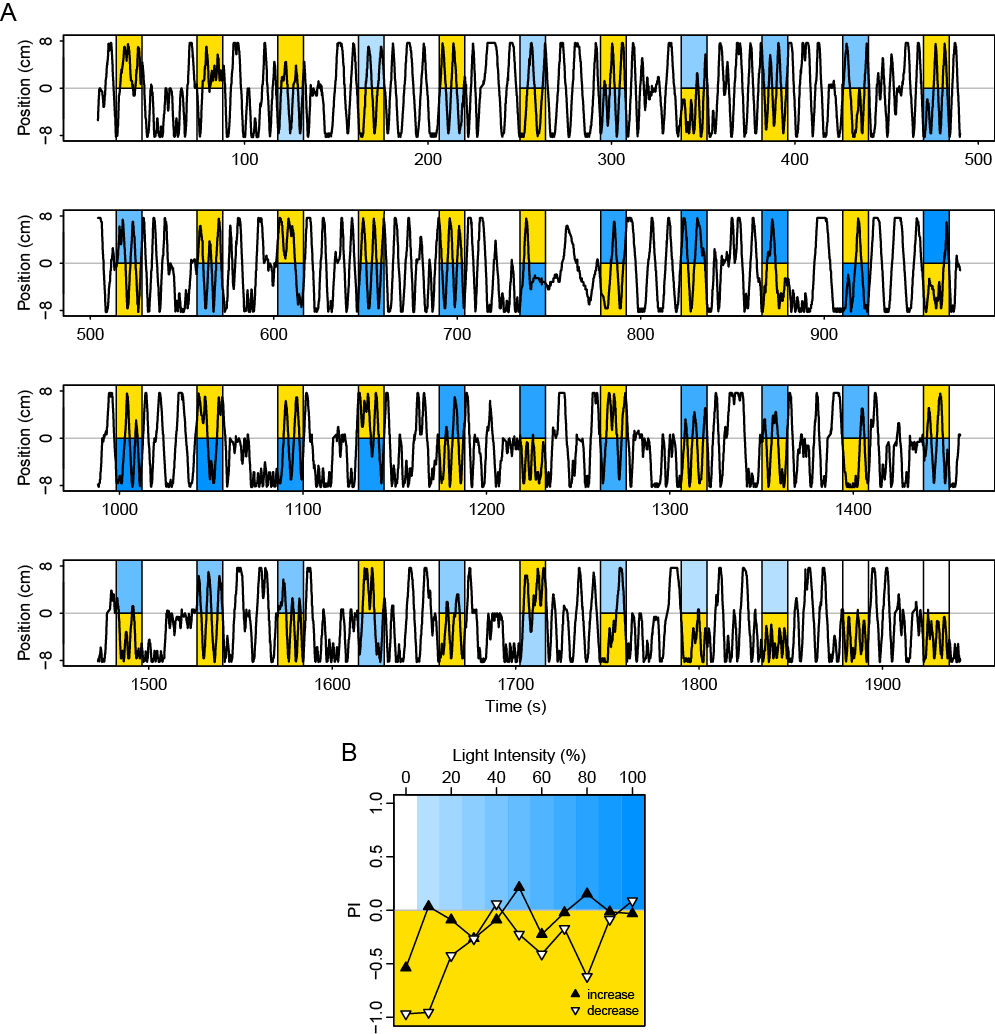


Figure S 14: (A) Example trace from a bee that underwent light intensity preference protocol with yellow light at 100% as reference and blue light at increasing or decreasing decades of percentage as test light. To avoid bias due to starting position, each light intensity configuration was presented twice in a row, with the test light either at the bee-side or the opposite side of the chamber. (B) Preference Index achieved in the different light intensity configurations. The bee displayed equal preference for blue and yellow lights when both were at 100% intensity. Black triangles pointing upwards represent series were intensity of test light was increased, while white triangles pointing downwards represent series were intensity was decreased.


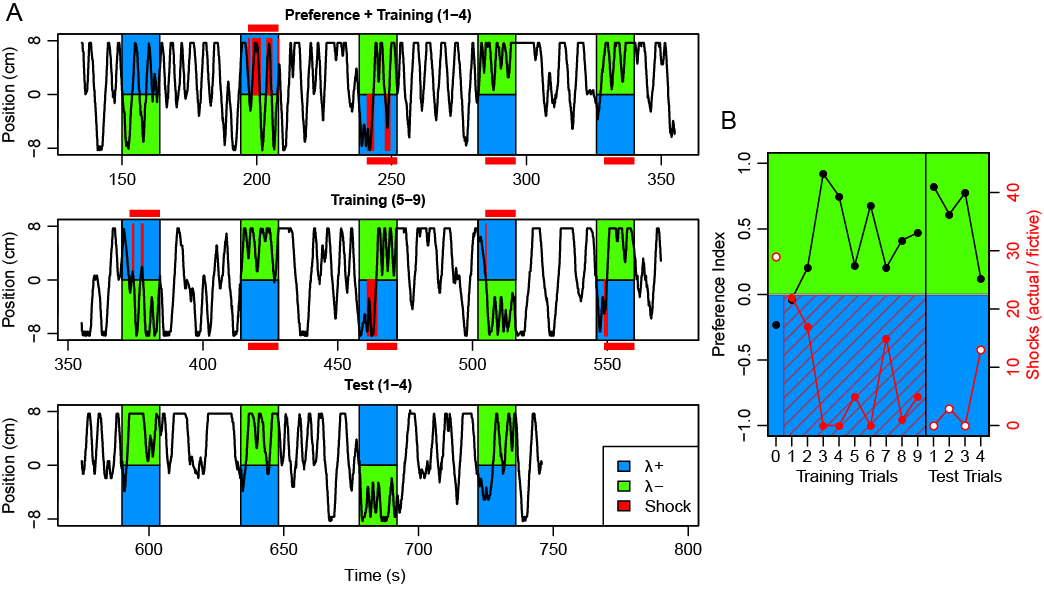


Figure S 15: (A) Example trace of bee conditioned with intensity corrected lights (zero preference intensity levels obtained in experiment 3). Blue light field was reinforced while green was safe. The first trial was unreinforced to establish a preference baseline. Red horizontal bars indicate shock availability of training trials. (B) Bee learned to avoid the shock-paired blue side, and changed its preference towards the green light field during the training. The bee continued this behavior in the test period, demonstrating stable short-term memory. Consequently, the bee received only a low nr of shocks after the second training trial.


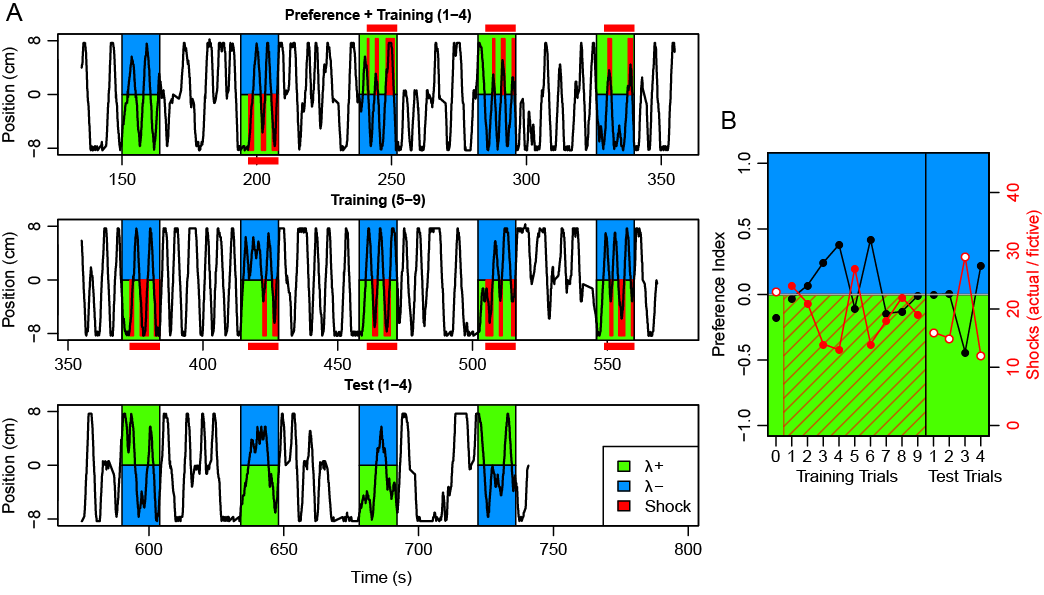


Figure S 16: (A) Example trace of bee conditioned with intensity corrected lights (zero preference intensity levels obtained in experiment 3). Green light field was reinforced while blue was safe. The first trial was unreinforced to establish a preference baseline. Red horizontal bars indicate shock availability of training trials. (B) Bee failed to learn the green light as bad, and did not alter its preference away from the green light field during the training. Consequently, the bee received a high nr of shocks throughout the training phase, and displayed no change in preference for the test phase.


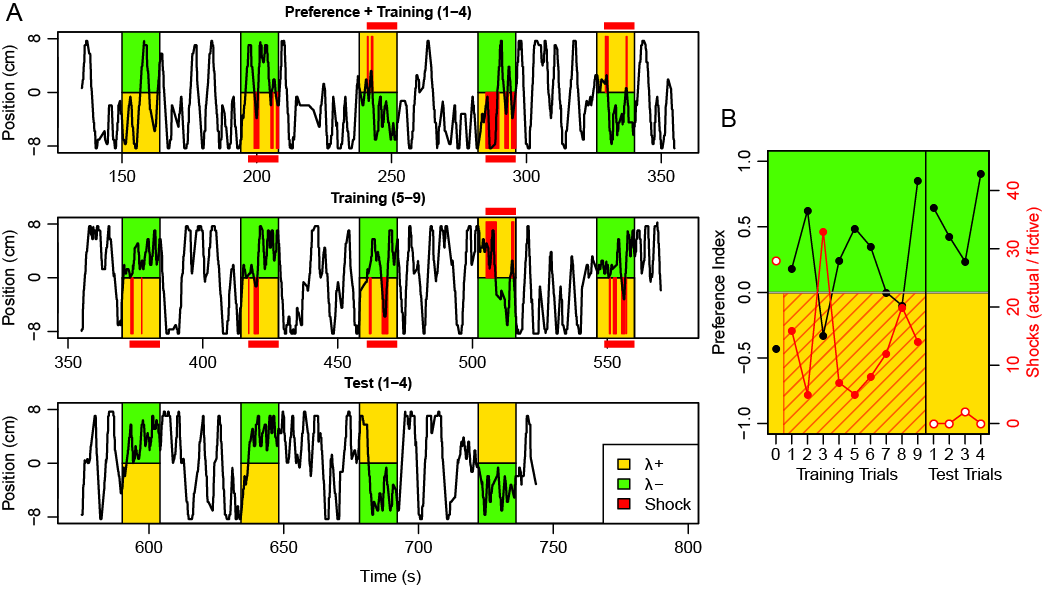


Figure S 17: (A) Example trace of bee conditioned with intensity corrected lights (zero preference intensity levels obtained in experiment 3). Yellow light field was reinforced while green was safe. The first trial was unreinforced to establish a preference baseline. Red horizontal bars indicate shock availability of training trials. (B) Bee learned to avoid the shock-paired yellow side, and changed its preference towards the green light field during the training. The bee continued this behavior in the test period, demonstrating stable short-term memory. Consequently, the bee received relatively few shocks throughout the second half of the training phase.


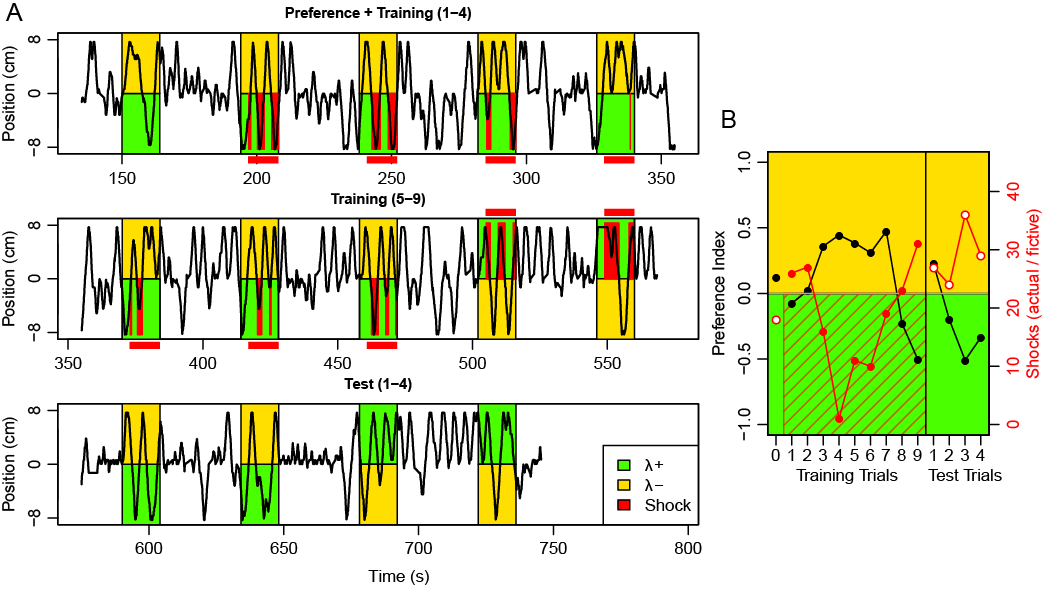


Figure S 18: (A) Example trace of bee conditioned with intensity corrected lights (zero preference intensity levels obtained in experiment 3). Green light field was reinforced while yellow was safe. The first trial was unreinforced to establish a preference baseline. Red horizontal bars indicate shock availability of training trials. (B) Bee failed to learn the green light as bad, and did not alter its preference away from the green light field during the training. Consequently, the bee received a high nr of shocks throughout the training phase, and displayed no change in preference for the test phase.
